# *ESTIM*ation of the *ABiLi*ty of prophylactic central compartment neck dissection to modify outcomes in low-risk differentiated thyroid cancer: a prospective randomized trial

**DOI:** 10.1186/s13063-023-07294-0

**Published:** 2023-04-28

**Authors:** Dana Hartl, Yann Godbert, Xavier Carrat, Stéphane Bardet, Audrey Lasne-Cardon, Pierre Vera, Elena Ilies, Slimane Zerdoud, Jérôme Sarini, Mohamad Zalzali, Luigi La Manna, Olivier Schneegans, Antony Kelly, Philppe Kauffmann, Patrice Rodien, Laurent Brunaud, Solange Grunenwald, Elie Housseau, Salim Laghouati, Nathalie Bouvet, Elodie Lecerf, Julien Hadoux, Livia Lamartina, Martin Schlumberger, Isabelle Borget

**Affiliations:** 1 Gustave Roussy, 114 rue Edouard Vaillant, 94805 Villejuif Cedex, France; 2 Institut Bergonié, 229 Cr de l’Argonne, 33076 Bordeaux Cedex, France; 3 Centre François Baclesse, 3 Av. du Général Harris, 14000 Caen, France; 4 Centre Henri Becquerel, 1 Rue d’Amiens, 76038 Rouen, France; 5 Institut Universitaire du Cancer de Toulouse, 1 avenue Irène Joliot-Curie, 31059 Toulouse Cedex 9, France; 6 Institut Godinot, 1 Rue du Général Koenig, 51100 Reims, France; 7 ICANS Institut de Cancérologie Strasbourg Europe, 17 Rue Albert Calmette, 67200 Strasbourg, France; 8 Centre Jean Perrin, 58, rue Montalembert, 63011 Clermont-Ferrand Cedex 01, France; 9 Centre Hospitalier Universitaire d’Angers, 4 Rue Larrey, 49100 Angers, France; 10 Centre Hospitalier Régional et Universitaire de Nancy, Rue du Morvan, 54000 Nancy, France; 11 Centre Hospitalier Universitaire de Toulouse, 2 Rue Charles Viguerie, 31300 Toulouse, France; 12 Centre Hospitalier de Hagenau, 64 Av. du Professeur René Leriche, 67500 Haguenau, France

**Keywords:** Thyroid cancer, Papillary, Low risk, Prophylactic neck dissection

## Abstract

**Background:**

Prophylactic central neck dissection in clinically low-risk cT1bT2N0 papillary thyroid carcinoma is controversial, due to a large number of conflicting retrospective studies, some showing an advantage in terms of locoregional recurrence, others showing no advantage. These previous studies all show high rates of excellent response. We aim to demonstrate the non-inferiority of thyroidectomy alone as compared to total thyroidectomy with prophylactic central neck dissection in conjunction with adjuvant RAI 30 mCi with rTSH stimulation in terms of excellent response at 1 year.

**Trial design and methods:**

Prospective randomized open multicenter phase III trial including patients with 11–40-mm papillary thyroid carcinoma (Bethesda VI) or suspicious cytology (Bethesda V) confirmed malignant on intra-operative frozen section analysis, with no suspicious lymph nodes on a specialized preoperative ultrasound examination. Patients will be randomized 1:1 into two groups: the reference group total thyroidectomy with bilateral prophylactic central neck dissection, and the comparator group total thyroidectomy alone. All patients will receive an ablative dose of 30mCi of radioactive iodine (RAI) within 4 months of surgery. The primary outcome is to compare the rate of excellent response at 1 year after surgery between the groups, as defined by an unstimulated serum thyroglobulin (Tg) level ≤ 0.2 ng/mL with no anti-Tg antibodies, an normal neck ultrasound and no ectopic uptake on the post-RAI scintiscan. Non-inferiority will be demonstrated if the rate of patients with excellent response at 1 year after randomization does not differ by more than 5%. Setting the significance level at 0.025 (one-sided) and a power of 80% requires a sample size of 598 patients (299 per group). Secondary outcomes are to compare Tg levels at 8 +/− 2 postoperative weeks, before RAI ablation, the rate of excellent response at 3 and 5 years, the rate of other responses at 1, 3, and 5 years (biochemical incomplete, indeterminate, and structurally incomplete responses), complications, quality of life, and cost-utility.

**Discussion (potential implications):**

If non-inferiority is demonstrated with this high-level evidence, prophylactic neck dissection will have been shown to not be necessary in clinically low-risk papillary thyroid carcinoma.

**Trial registration:**

NCT 03570021. June 26,2018

**Supplementary Information:**

The online version contains supplementary material available at 10.1186/s13063-023-07294-0.

## Administrative information

**Table:** 

Title	**ESTIM**ation of the **AB**i**L**ity of prophylactic central compartment neck dissection to modify outcomes in low-risk differentiated thyroid cancer: A Prospective Randomized Trial **Abbreviated Title of Protocol (Acronym): ESTIMABL 3**
Trial registration	NCT 03570021 https://clinicaltrials.gov/ct2/show/NCT03570021?term=nct03570021&draw=2&rank=1 French trial registration number 2017-A01779-44
Protocol version	Protocol version 2.3, October 22, 2021 (submitted to Clinical Trials.gov as version 2B on December 20, 2021)
Funding	Funded by a grant from the French National Cancer Institute, PHRC-K15-182
Author details	Dana M. Hartl, MD PhD Principal Investigator Isabelle Borget, PharmD Methodologist and statistician Nathalie Bouvet Forteau Data manager Salim Laghouati, MD Pharmacovigilance Elodie Lecerf Clinical Research Assistant, Promotor Yann Godbert, Xavier Carrat, Stéphane Bardet, Audrey Lasne-Cardon, Pierre Vera, Elena Ilies, Slimane Zerdoud, Jérôme Sarini, Mohamad Zalzali, Luigi La Manna, Olivier Schneegans, Antony Kelly, Philppe Kauffmann, Patrice Rodien, Laurent Brunaud, Solange Grunenwald, Elie Housseau Trial co-investigators Livia Lamartina, MD PhD ; Julien Hadoux, MD PhD Co-investigators, co-coordinators TuThyRef network Martin Schlumberger, MD PhD Founder TuThyRef network
Name and contact information for the trial sponsor	Gustave Roussy 114 rue Edouard Vaillant 94805 Villejuif Cedex France Contact : Dr Dana M. Hartl, MD PhD dana.hartl@gustaveroussy.fr
Role and responsibilities of sponsor and funder	Study sponsor: Organization of data collection, management of clinical research assistants Study funder: approval of study design prior to funding The interpretation of data, writing of the report and communication and publication of the results are under the responsibility of the principal investigator and methodologist. The sponsor and funder do not have oversight of data interpretation or publication.
Role and responsibilities of committees	Coordinating center: Gustave Roussy (also promotor), coordination of participating centers, communication with the principal investigators and research assistants of each participating center Data management committee, data management team from Gustave Roussy: oversight of correct and complete reporting

## Introduction

### Background and rationale

For most cancers, the extent of metastasis to regional lymph nodes is a prognostic factor, and prophylactic treatment of lymph node basins for patients with no preoperative evidence of nodal disease (cN0) is often recommended. For differentiated thyroid cancer, however, the prognostic role of prophylactic central compartment neck dissection (PND) associated with total thyroidectomy for patients cN0 constitutes a major controversy for these tumors with an increasing incidence, but a very low mortality rate.

Those in favor of PND for whom it is a standard of care cite the low-level evidence suggesting

- Improved recurrence-free survival (retrospective case series), [1–3]
- A higher rate of recurrence in the presence of lymph node metastases (in some retrospective studies) and the usefulness of a complete staging in the neck to stratify for radioactive iodine treatment, [4, 5]
- The technical difficulty of performing a reintervention in the central compartment secondarily, and
- The absence of increased permanent complications of PND (in experienced hands). [3]

Opponents of PND cite the low-level evidence suggesting

- No effect on oncologic outcomes with PND (retrospective case series), [6, 7]
- The low rate of recurrence and mortality even without PND, [8]
- The efficacy of radioactive iodine to treat microscopic nodal metastases,
- A higher risk of complications (hypoparathyroidism and vocal fold paralysis) as compared to -thyroidectomy alone, [9]
- The feasibility of reoperation in the central compartment if needed with a relatively low risk of complications (in experienced hands), [10, 11] and
- An increase in cost with PND. [12]

Despite the low-level conflicting evidence, different professional societies in several countries have published recommendations for or against the routine use of PND, with consequences for patients, physicians, and healthcare providers [13–19]. The French Society of Otolaryngology Head and Neck Surgery recommends PND [15] whereas the Francophone Association of Endocrine Surgery does not recommend it [19].

This study aims to provide level I evidence with a multicenter prospective open randomized non-inferiority trial comparing bilateral PND with total thyroidectomy to total thyroidectomy alone (and adjuvant radioactive iodine in both groups) for low-risk papillary thyroid cancer patients cT1bT2N0 in terms of the rate of complete remission at 1 year after randomization. We hypothesize that thyroidectomy alone is not inferior to thyroidectomy with PND by more than 5% at 1 year.

Differentiated thyroid carcinoma is the most common endocrine malignancy, with an increasing incidence in part related to an improvement in screening for small tumors by neck ultrasound. It accounts for 80% of all thyroid cancers and ranks as the sixth most common cancer in females in incidence, with approximately 10,000 new cases per year in France [20, 21]. The high disease-specific survival rates favor optimizing treatment and follow-up to minimize complications and overtreatment. Macroscopic lymph node metastases detected on preoperative ultrasound (cN1) are known to increase regional recurrence, with the risk of recurrence related to the size and number of metastatic nodes and the presence or absence of extranodal tumor extension;[22, 23] in this case a therapeutic central compartment neck dissection is currently recommended with no controversy [13].

However, the role of systematic prophylactic neck dissection (PND) in the absence of suspected neck metastases on preoperative ultrasound (cN0) remains controversial. For example, guidelines from the American Thyroid Association (ATA),[13] The European Society of Endocrine Surgeons,[24] the German Association of Endocrine Surgeons,[16] and the Francophone Association of Endocrine Surgery [19] do not recommend PND. The British Thyroid Association [17] and the Japanese Association of Endocrine Surgeons [14] recommend PND in certain cases (large tumors, older patients, and extrathyroidal extension, for example). Finally, the 2012 guidelines from the French Society of Otolaryngology Head and Neck Surgery recommend systematic PND [15].

All of these recommendations are grade C (“expert opinion”) due to the lack of high-level evidence in the field. The proponents of systematic PND have underlined its potential effect on recurrence-free survival and disease-specific survival, shown in some retrospective studies, [1–3] but not found in several meta-analyses [6, 25, 26]. The opponents of systematic PND have emphasized that the prognostic role of occult metastases has not been demonstrated and that radioactive iodine is effective in treating micrometastases [27, 28]. PND may also involve greater morbidity in terms of transient hypoparathyroidism [9]. A higher level of evidence is needed to optimize treatment of these low-risk tumors, which make up the majority of differentiated thyroid cancers.

Current practice is based on conflicting data from studies with low-level evidence and due to the absence of a randomized trial, conflicting recommendations as to the optimal management of low-risk thyroid cancer will prevail [29].

A PubMed® literature search using the terms “thyroid cancer prophylactic neck dissection” found 512 results (accessed January 2023). Among these, Table 1 resumes those with the highest level evidence (meta-analysis of randomized trials, prospective randomized trials, and meta-analyses of retrospective studies). The majority of these studies do not show a difference in locoregional recurrence between total thyroidectomy with PND versus total thyroidectomy alone.

**Table 1:** Summary of publications with the highest level of evidence

**Study**	**Design**	**Number of patients**	**Results concerning recurrence rates**	**Other oncologic results**	**Complication rates**
Sanabria A et al Ann Surg 2022 [30]	Meta-analysis of 5 randomized studies	763 409 PND 354 TT	No difference in structural recurrence rate 2.5% vs 2.7%	No difference in biochemical recurrence rate	No difference in rate of permanent hypoPTH
Ahn JH et al Surgery 2022 [31]	Propsective randomized single center	101 51 PND 50 TT	No difference in structural recurrence rates or successful ablation		No difference in complication rate
Sippel R et al Ann Surg 2020 [32]	Propsective randomized single center	60 30 PND 30 TT	No difference in stimulated Tg levels at 6 weeks or 1 year		
Lee DY et al JCEM 2015 [33]	Propsective randomized single center	257 153 PND 104 TT	No difference in LRR 3.3% vs 3.9%	No difference in number of patients treated with RAI	Higher rates of temporary hypoPTH (*p*=0.043)
Viola D et al JCEM 2015 [34]	Propsective randomized single center	181 93 PND 88 TT	No difference in LRR (7.5% vs 8.0%)	More RAI in TT alone group (*p*=0.002)	Higher rate of permanent hypoPTH in PND group (*p*=0.02)
Chen L et al World J Surg 2018 [26]	Meta-analysis of retrospective studies	18,376 11,098 PND 5583 TT	Lower LRR in PND group 2.52% vs 4.59% (OR=0.65)		Higher rates of temporary (OR=2.23)and permanent hypoPTH (OR=2.22)and temporary VFP (OR=2.03)
Liang J et al Acta Otorhino Ital 2017 [35]	Meta-analysis of retrospective studies	6823 3312 PND 3511 TT	Lower LRR in PND group (*p*<0.01)		Higher rate of temporary (*p*<0.01) and permanent (*p*<0.01) hypoPTH and temporary VFP (*p*=0.023) in PND group
Zhao W et al Ann Surg Oncol 2016 [36]	Meta- analysis of retrospective studes	4437 1969 PND 2468 TT	Lower LRR in PND group 1.1% vs 3.4% (*p*=0.002)	More RAI in PND group 74.6% vs 59.9% (OR 1.20)	Higher rates of temporary (*p*<0.00001) and permanent (*p*=0.03) hypoPTH
Wang TS et al Ann Surg Oncol 2013 [6]	Meta- analysis of retrospective studes	1740 745 PND 995 TT	No difference in LRR		No difference in rates of permanent complcations
Lang BH et al Thyroid 2013 [7]	Meta-analysis of retrospective studies	3331 1592 PND 1739 TT	Lower LRR in PND group 4.7% vs 8.6% (OR=0.65)	More RAI in PND group 71.7% vs 53.1% (OR=2.60)	Higher rate of temporary hypoPTH PND group 26% vs 10.8% (OR=2.56)
Zetoune T et al Ann Surg Oncol 2010 [37]	Meta-analysis of retrospective studies	1264 396 PND 868 TT	No difference in LRR (2.02% vs 3.92%)		

Abbreviations: *PND* prophylactic central neck dissection, *TT* total thyroidectomy without prophylactic neck dissection, *LRR* locoregional recurrence rate, *RAI* radioactive iodine, *hypoPTH* hyypoparathyrodism, *Tg* thyroglobulin, *VFP* vocal fold paralysis, *OR* odds ratio

Our study differs from these published studies in the following ways:

- Our study includes only tumors ≥11 mm (microcarcinomas are not eligible), whereas all of the studies cited in Table 1, with the exception of the study by Sippel et al. [32], included a large proportion of microcarcinomas (in Ahn et al. [31] for example, 90% of the tumors were T1 with a mean tumor size of 1.1 +/− 0.6 cm);
- Thyroglobulin measurements will be evaluated before and after the administration of RAI to evaluate the effect of RAI ablation and eliminate this bias found in most of these studies in which outcomes were evaluated after RAI ablation in most or all patients;
- Finally, our study is designed with a non-inferiority margin of 5% and an alpha level set at 0.025, whereas the study by Viola et al. [34] is significant but with a non-inferiority margin of 15% and an alpha of 0.05.

### Rationale: choice of comparators

In France, total thyroidectomy with prophylactic central compartment (level VI) neck dissection as defined by the American Thyroid Association [38] is standard treatment recommended by the French Society of Otolaryngology Head and Neck Surgery,[15] whereas total thyroidectomy alone without neck dissection is recommended as standard treatment by the Francophone Association of Endocrine Surgery [19].

A simulation by the American Thyroid Association, hypothesizing a 25% difference in significant oncologic events at 7 years, with a statistical power of 80%, concluded that a randomized trial was “not readily feasible” due to the need to randomize 5840 patients [39]. This implies that both therapeutic strategies may indeed be equivalent within a small percentage, and we have therefore designed our study with a *non-inferiority statistical design with a surrogate endpoint*. This methodology (non-inferiority trial) has been clearly validated in these low-risk patients by two previous studies carried out by the same French thyroid network (TuThyRef) and published in the New England Journal of Medicine, ESTIMABL[40] and ESTIMABL2 [41]. The surrogate endpoint in the present study is the rate of excellent response as defined by the ATA,[13] or complete remission, at 1 year.

If our study confirms the non-inferiority of total thyroidectomy alone, prophylactic central compartment neck dissection could be abandoned for these low-risk patients without taking undue oncologic risks. Secondary endpoints may also show a benefit in terms of patient quality of life and of cost-utility analysis with a total thyroidectomy alone. Patients, physicians, and healthcare providers would all benefit from less-extensive surgery.

### Objectives

#### Primary objective

To assess the non-inferiority of total thyroidectomy alone as compared to total thyroidectomy with bilateral prophylactic central compartment neck dissection in terms of the rate of complete remission (excellent response) at 1 year after randomization, for differentiated thyroid cancer cT1bT2N0. We hypothesize that the rate of complete remission after thyroidectomy alone is not inferior to the rate of complete remission after thyroidectomy with PND by more than 5% at 1 year.

The primary criterion is the rate of patients *in complete remission* (excellent response) at 1 year after randomization (8+/2 months post-^131^I) as defined by the presence of all 3 criteria:

- Normal whole body scan (SPECT-CT) performed after the administration of 30 mCi (1.1 GBq) of 131I administered within 2–4 months following surgery,
- Normal neck ultrasound 8+/−2 months after the ^131^I
- Unstimulated ultrasensitive thyroglobulin while on L-thyroxine treatment (usTg/LT4) ≤ 0.2 ng/mL) without anti-Tg antibodies (TgAb) 8+/−2 months after administration of ^131^I

In this network’s previous study, 94% of the low-risk patients (including 12% T2N0) were in complete remission 1 year after surgery and administration of 131I after rhTSH [40]. In a similar prospective multicentre trial, 90.2% of the low-risk patients, including patients T1–T3 N0–N1 with or without central compartment neck dissection, were in complete remission 1 year after surgery and administration of 131I after rhTSH [42]. For low-risk patients showing an excellent response after treatment, the risk of recurrence is estimated to be 2–3% [4].

#### Secondary objectives

To compare total thyroidectomy and radioactive iodine (^131^I) to total thyroidectomy with bilateral prophylactic central compartment neck dissection and 131I in terms of:

- Thyroglobulin levels after surgery alone (usTg/T4) measured while on T4 treatment, 8+/−2 weeks postoperatively, before stimulation and administration of radioactive iodine
- Percent of patients in complete remission (excellent response) at 3 and 5 years after randomization, as defined by a normal neck ultrasound and usTg/LT4 ≤ 0.2 ng/m. The endpoint of 5 years reflects the data from a prospective multicenter study of 715 patients reporting that 81% of recurrences occurred within 5 years,[43] and from a retrospective study of 1020 patients followed for 10 years reporting that all structural recurrences occurred within 8 years, with 77% occurring within 5 years [44].
- Percent of patients at 1, 3, and 5 years after randomization with structural incomplete response in the neck defined by a malignant lesion in the neck detected by ultrasound and confirmed by cytology (and/or Tg in the needle washout fluid >10ng/ml). Cytology will be mandatory for suspicious lesions measuring 8mm or more in the smallest diameter; lesions with suspicious features on ultrasound but measuring <8 mm may undergo cytology at the discretion of the center’s principal investigators.
- Percent of patients at 1, 3, and 5 years after randomization with biological incomplete response defined by a normal neck ultrasound and absence of disease detected on other conventional or metabolic imaging (^131^I, ^18^FDG-TEP), if performed, associated with a serum Tg/LT4>0.2 ng/ml
- Percent of patients at 1, 3, and 5 years after randomization with an indeterminate response defined by a suspicious lesion on neck ultrasound without cytological proof of disease and/or detection of TgAb
- Percent of patients at 1, 3, and 5 years after randomization with diagnosis of distant metastases on metabolic imaging (^131^I, ^18^FDG-TEP) or cross-sectional imaging, and confirmed cytologically (except for metastases with ^131^I uptake) or with repeat imaging at 6 months (if cytology not possible)
- Percent of patients at 1, 3, and 5 years after randomization having undergone further treatment (surgery or 2nd therapeutic administration of ^131^I, number of retreatments per patient and indication for each retreatment)
- At 1 year: percent of patients with persistent hypoparathyroidism with supplementation and/or persistent vocal fold paralysis; subjective dysphonia (Voice Handicap Index)[45] and dysphagia (SWAL-QOL)[46] (questionnaires in their validated French versions) compared between groups after randomization: Quality of life SF36[47], EuroQol EQ-5D,[48] Anxiety (State-Trait Anxiety Inventory-STAI)[49]
- Cost-utility analysis.

### Trial design

Prospective randomized open phase III non-inferiority trial in patients with cT1bT2N0 [50] papillary thyroid carcinoma comparing: total thyroidectomy alone (experimental group) versus total thyroidectomy with prophylactic neck dissection (PND) (reference group).

*Group 1 (reference group)*: total thyroidectomy with bilateral prophylactic central compartment (level VI) neck dissection

*Group 2 (“experimental” group)*: total thyroidectomy alone without neck dissection.

Before surgery, the patients will first be pre-registered (included) to check that the thyroid nodule is classified cT1bT2N0 and the FNAB cytology is classified type 5 or 6 according to the Bethesda classification [51].

Surgery must be performed within 4 months of inclusion.

- For patients with *FNAB cytology Bethesda 6 “papillary carcinoma”*, inclusion and randomization will we performed preoperatively.
- For patients with *FNAB cytology Bethesda 5 “suspicious cytology”*, randomization (and validation of the inclusion) will then be performed in the operating room, after confirmation of malignancy by intra-operative frozen section analysis. For these patients, randomization will be performed online or by fax with the Trial Master program.

All patients will have Tg/LT4 measured 8 +/−2 weeks postoperatively, *before stimulation with recombinant human thyrotropin (rhTSH).*

All patients will receive, 2–4 months postoperatively, 30 mCi (1.1 GBq) ^131^I after stimulation with human recombinant thyrotropin (rhTSH) and undergo scintiscan with SPECT-CT. Neck ultrasound will be performed at the time of ^131^I administration (standard of care).

Patients will be evaluated at 8 +/−2 months post-iodine (8–14 months postoperatively or “1 year”) then yearly with neck ultrasound, unstimulated ultrasensitive thyroglobulin (usTg/LT4), and anti-Tg antibodies.

## Methods: participants, interventions, and outcomes

### Study setting

This is a multicenter study involving only specialized university hospitals and comphrehensive cancer centers in France. The list of study sites can be obtained by contacting the principal investigator or data manager at Gustave Roussy.

### Eligibility criteria

#### Inclusion criteria

1. Thyroid nodule measuring *11–40 mm* on ultrasound (*cT1bT2*)
  AND with fine-needle aspiration biopsy (FNAB) cytology in favor of “papillary thyroid carcinoma” (*Type 6 according* to the Bethesda classification (Appendix 2)
*OR* with FNAB cytology “suspicious for malignancy” (*Type 5* according to the Bethesda classification). In this latter case, randomization will be performed if confirmation of papillary carcinoma on intra-operative frozen section analysis
2. cN0: absence of lymph nodes suspicious for malignancy on preoperative ultrasound performed by the center’s designated radiologists according to a standardized report[52]
3. Absence of a medical contraindication to performing a total thyroidectomy with or without bilateral prophylactic neck dissection of the central compartment
4. Women of childbearing potential should have a negative pregnancy test (serum or urine) before any radioiodine administration. Sexually active patients must agree to use an effective method of contraception or to abstain from sexual activity during the study and for at least 6 months after last dose of radioiodine.
5. Patient affiliated to a social security regimen or beneficiary of such regimen
6. Patients age ≥ 18 years old, French-speaking
7. Patients should understand, sign, and date the written informed consent form prior to any protocol-specific procedures. Patients should be able and willing to comply with study visits.

#### Non-inclusion criteria

1. Tumors > 40 mm (cT3) or ≤ 10 mm
2. Tumors with extrathyroidal extension suspected or obvious on the preoperative work-up or intra-operatively (cT3T4)
3. Metastatic neck lymph nodes or suspicious neck nodes on preoperative ultrasound (cN1); for suspicious nodes, FNAB cytology and thyroglobulin assay on the needle washout fluid will be performed
4. Metastatic neck lymph nodes found during the thyroidectomy and confirmed with intra-operative frozen section analysis
5. Medullary thyroid carcinoma on FNAB cytology and/or with basal serum calcitonin >50 pg/ml
6. Preoperative or intra-operative suspicion of non-papillary thyroid carcinoma or aggressive histopathological subtype or poorly differentiated carcinoma
7. Distant metastases (M1) apparent preoperatively (found due to symptoms or fortuitously; no specific preoperative work-up will be performed, however, in accordance with current clinical practice)
8. Recurrent nerve paralysis visualized on systematic preoperative laryngoscopy and/or abnormal preoperative serum calcium
9. Pregnant or breast feeding women
10. Participation in another therapeutic clinical trial within 1 year from study entry
11. Patient under guardianship or deprived of their liberty by a judicial or administrative decision or incapable of giving their consent

### Criteria for eligibility of participating centers

The surgeons, endocrinologists, radiologists, and nuclear medicine physicians participating in this clinical trial are practitioners exercising medicine in either a university hospital or a comprehensive cancer center in France that belongs to the ENDOCAN-TuThyRef network, a network for treatment of thyroid cancer sponsored and financed by the French National Cancer Institute. All participants have expertise in treating thyroid cancer. Furthermore, the participating surgeons routinely perform complete central neck dissections and were chosen to participate in this study due to a homogenous technique among these surgeons [53].

### Who will take informed consent?

Prior to registration in the trial, written informed consent is obtained from the patient during a consultation. The surgeon, endocrinologist, or nuclear medicine physician informs the patient and obtains their informed consent. Two identical consent forms are signed with one of the original forms retained by the patient, the other retained by the investigators. The patient information and consent form can be found in Annex 1. Additional informed written consent is obtained at the same time for use of biological specimens for research, in accordance with the articles L1211-1 to 9 of the French Public Health Code.

## Interventions

### Explanation for the choice of comparators

The primary objective of this study is to assess the non-inferiority of total thyroidectomy alone as compared to total thyroidectomy with bilateral prophylactic central compartment neck dissection in terms of the rate of complete remission (excellent response) at 1 year after randomization, for differentiated thyroid cancer cT1bT2N0.

Group 1 (reference group): total thyroidectomy with bilateral prophylactic central compartment (level VI) neck dissection as defined by the American Thyroid Association [38]. This is a standard treatment recognized by the French Society of Otolaryngology Head and Neck Surgery [15]. The number of lymph nodes resected, the number of metastatic nodes, their size, and the presence or absence of extranodal spread will be recorded. Due to the large natural variability of the number of lymph nodes retrieved in a PND, [53] no patient will be excluded on the basis of number of lymph nodes.

Group 2 (“experimental” group): total thyroidectomy alone without neck dissection. This is recognized as a standard treatment by the Francophone Association of Endocrine Surgery [19].

### Intervention description

#### Randomization of patients

- *Pre-registration (inclusion)* of eligible patients after signed informed consent.
  - Surgery must be performed within 4 months of pre-registration.
  - Quality of life (SF-36, EQ-5D, STAI) questionnaires will be completed by the patient within 1 month before the surgery.
  - Before surgery, the patients will first be pre-registered (included) to check that the thyroid nodule is classified cT1bT2N0 and the FNAB cytology is classified type 5 or 6 according Bethesda (Appendix 2).
- Randomization (and validation of the inclusion)

Randomization (and validation of the inclusion) will then be performed:

1. Before surgery for patients with malignant cytology (Bethesda 6)
2. OR in the operating room, after total thyroidectomy without any particular dissection of paratracheal spaces and after confirmation of malignancy by intra-operative frozen section analysis for patients with suspicious cytology (Bethesda 5).

**Fig. 1:**
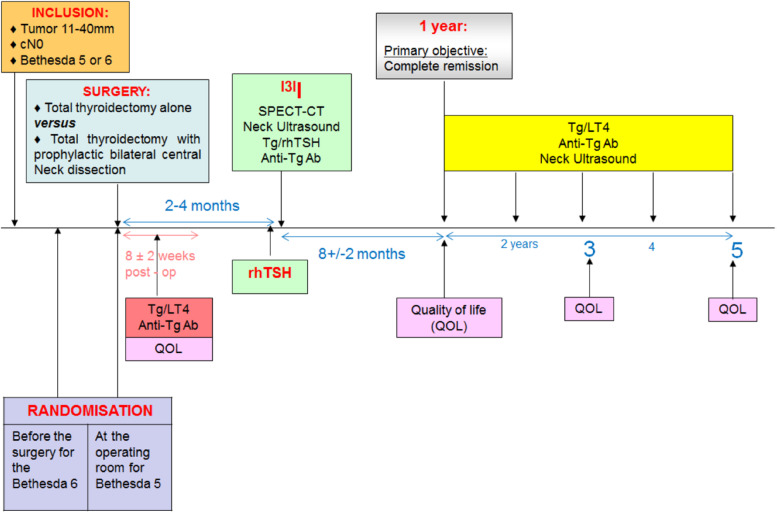
Diagram of the trial design

**Fig. 2:**
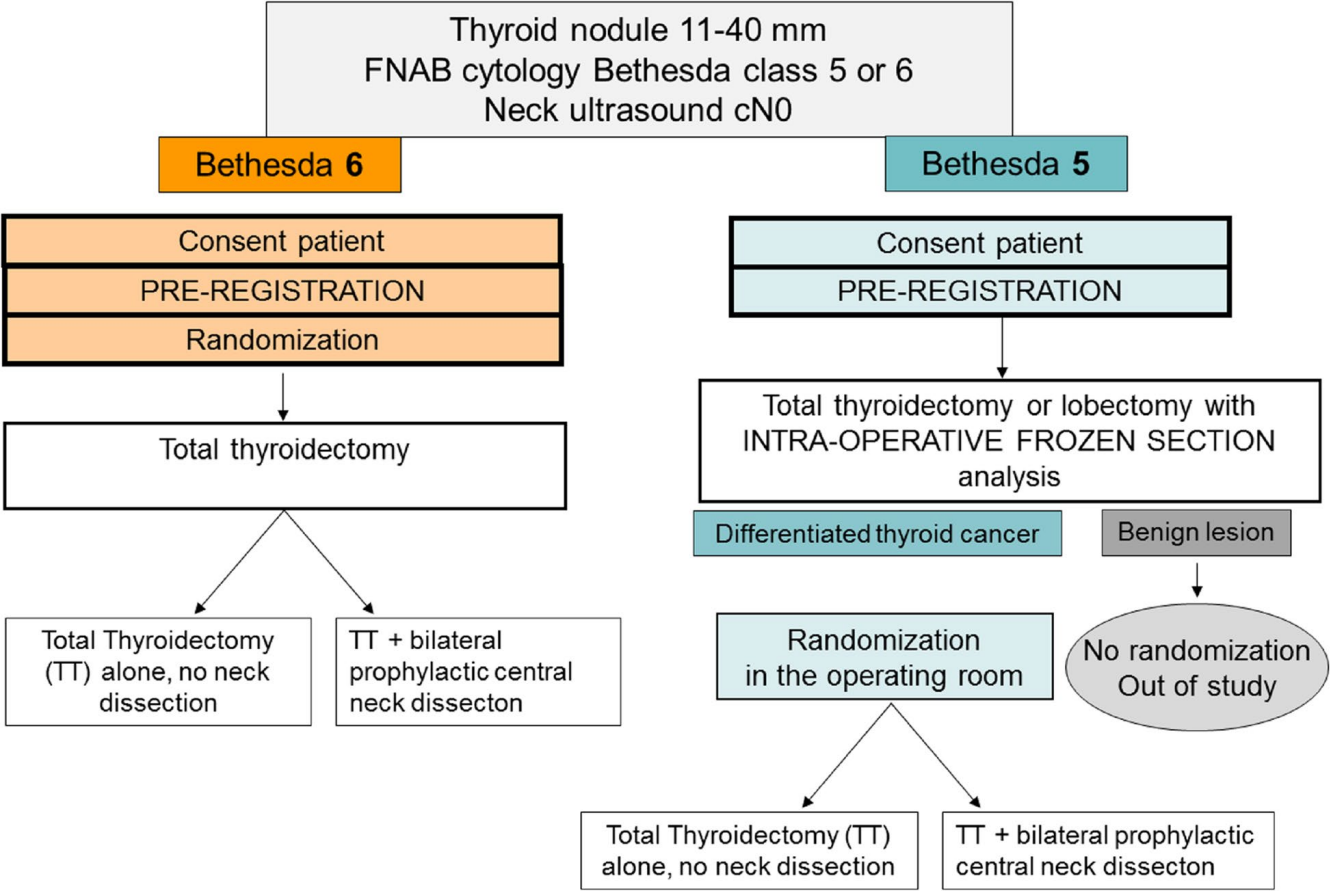
Diagram of randomization

For patients with a nodule Bethesda 5, the randomization form will incude a confirmation of malignancy of the intra-operative frozen section analysis for patients with suspicious cytology Figs. 1 and 2.

#### Treatments

##### Baseline assessment

- Inclusion and non-inclusion criteria and patient consent
- Neck ultrasound
- Laryngoscopy
- Serum calcium
- EQ-5D 3 Levels (Annex 2) [48]
- Quality of life (SF-36) (Annex 3) [47]
- Anxiety (State-Trait Anxiety Inventory) (Annex 4) [49]
- Subjective dysphonia and dysphagia (measured via the Voice Handicap Index (ANNEX 5) [45] and SWAL-QOL) (Annex 6)[46]

##### Surgery

- Group 1 (reference group): total thyroidectomy with bilateral prophylactic central compartment neck dissection
- Group 2 (“experimental” group): total thyroidectomy alone without neck dissection.

##### Radioactive iodine

All patients will have Tg/LT4 measured 8 +/− 2 weeks postoperatively, before stimulation with recombinant human thyrotropin (rhTSH).

Then, for all patients (whichever the group), radioactive iodine will be administered after 2 months but within 4 months postoperatively: 30 mCi (1.1 GBq) ^131^I after stimulation with human recombinant thyrotropin (rhTSH)

A ß-HCG test (serum or urine) will be performed before any radioiodine administration.

##### Follow-up

All patients will have a postoperative visit within 4 months of the surgery with the surgeon to record post-op complications.

For all patients, Tg/LT4 and anti-Tg antibodies (anti-Tg Ab) measured 8 +/−2 weeks postoperatively, before stimulation with recombinant human thyrotropin (rhTSH).

At 8 +/− 2 weeks post-op before 131I:

-Quality of life (SF-36) [47]
-EQ-5D 3 Levels [48]
-Anxiety (State-Trait Anxiety Inventory) [49]
-Subjective dysphagia (measured via the SWAL-QOL questionnaire) [46]

For all patients (after 2 months but within 4 months postoperatively at the time of 131I administration:

- Stimulated usTg (Tg/rhTSH), anti-Tg antibodies (anti-Tg Ab)
- Neck ultrasound
- Whole body scintiscan with SPECT performed 2–5 days after the RAI administration
- Quality control of total thyroidectomy will be ensured by calculation of the % of 131I uptake, as an estimate of the size of thyroid remnant.

Further follow-up consists in:

- 1 year after surgery (8 +/− 2 months after 131I) (primary endpoint): usTg on thyroxine treatment (usTg/LT4) with a standard ultrasensitive kit, anti-Tg Ab, neck ultrasound
- Yearly for 5 years (+/− 2 months after 131I): Tg/LT4, anti-Tg Ab, neck ultrasound
- If suspicious lesion (according to standardized criteria on ultrasound [52] > or = 8 mm (smallest dimension): indication for cytological examination by fine-needle aspiration biopsy (FNAB) and, for lymph nodes, for a determination the of level of thyroglobulin (Tg) in the needle washout fluid
- If a suspicious lesion <8mm is visualized on ultrasound (according to standardized criteria,[52] cytology with FNAB (and Tg in the needle washout fluid) will be performed upon decision of the center’s principal investigators

Functional evaluation and quality of life:

- Immediate postoperative complications (0–4 months after surgery) (hypoparathyroidism requiring supplementation, recurrent nerve paralysis visualized on systematic laryngoscopy)
- Complications at 1 year: treatment for persistent hypoparathyroidism, recurrent nerve paralysis visualized on systematic laryngoscopy performed at 1 year
- At 1 year after randomization: Subjective dysphonia (measured via the Voice Handicap Index [45]), Subjective dysphagia (measured via the SWAL-QOL questionnaire [46])
- Complete remission at 1, 3, and 5 years after randomization
- At 1, 3, and 5 years after randomization: Quality of life [SF-36 + EQ-5D], Anxiety (State-Trait Anxiety Inventory), all questionnaires in their validated French translation:
- Quality of life (SF-36) [47]
- EQ-5D 3 Levels [48]
- Anxiety (State-Trait Anxiety Inventory) [49]
- Subjective dysphonia (measured via the Voice Handicap Index, VHI) [45]
- Subjective dysphagia (measured via the SWAL-QOL questionnaire) [46]

##### Translational research

BRAF V600E mutational analysis will be performed on all primary tumors (corresponding to the surgical specimen recovered during the thyroidectomy) on formalin-fixed paraffin-embedded (FFPE) blocks or slides as a separate study not included herin.

### Criteria for discontinuing or modifying allocated interventions

Secondary exclusion will be performed for patients in whom final pathology (non-frozen section analysis but defintive pathological evaluation) does not confirm the presence of a differentiated thyroid cancer.

In no other instances does this study provide for discontinuing or modifying allocated interventions.

Reasons for withdrawal from the trial (study interventions and follow-up) may include:

- Lost to follow-up
- Withdrawal of consent
- Death

If a patient does not return for a scheduled visit, every effort will be made to contact them. In any case, every effort will be made to document the patient outcome and all attemps should be documented in the corresponding medical file. The investigator will inquire about the reason for withdrawal, request the patient to return for a final visit, if applicable, and follow up with the patient regarding any unresolved adverse events. The early termination final visit should include all assessments listed for the End of Study visit.

If the patient withdraws their consent for the study, no further study-specific evaluations will be performed, and no additional data will be collected. The sponsor may retain and continue to use any data and samples collected before such refusal except in case of patient opposition. Any opposition will be transmitted by the investigator to the sponsor without undue delay.

### Strategies to improve adherence to interventions

Locally in each center, follow-up visits will be programmed in advance and patients informed in advance of the follow-up program.

### Relevant concomitant care permitted or prohibited during the trial

Pregnancy is to be avoided before and for 6 months following the administration of ^131^I. Patients are counselled to use a method of contraception.

### Provisions for post-trial care

Patients will be followed as part of this clinical trial for a maximum of 5 years. After that period, routine follow-up as standard of care will be performed.

### SPIRIT Reporting guidelines

In this report, we have used the SPIRIT reporting guidelines [54].

### Outcomes: Primary criterion

The primary criterion is the rate of patients *in complete remission (excellent response) at 1 year after radomization* as defined by the presence of the 3 criteria:

- Normal whole body scan (SPECT-CT) at the time of administration of ^131^I [SPECT-CT will be performed 2–5 days after the administration of 30 mCi (1.1 GBq) of ^131^I after stimulation using injected recombinant human TSH (rhTSH)],
- Normal neck ultrasound at 8+/−2 months after ^131^I administration
- Unstimultaed ultrasensitive thyroglobuline while on L-thyroxine treatment (usTg/LT4) ≤ 0.2 ng/mL without anti-Tg antibodies (TgAb) at 8+/−2 months after ^131^I administration

### Outcomes: Secondary criteria

#### Oncologic secondary criteria

- Thyroglobulin levels after surgery alone (ultrasentive thyroglobuliin, usTg/T4) measured while on T4 treatment, 8 +/−2 weeks postoperatively, before stimulation or administration of radioactive iodine
- Percent of patients in complete remission (excellent response) at 3 and 5 years after randomization, as defined by negative imaging and either unstimultaed ultrasensitive thyroglobulin while on L-thyroxine treatment (usTg/LT4) ≤ 0.2 ng/mL) without anti-Tg antibodies (TgAb) or TSH-stimulated Tg<1ng/mL

The endpoint of 5 years reflects the data from a prospective multicenter study of 715 patients [Brassard M et al J Clin Endocrinol Metab 2011] reporting that 81% of recurrences occurred within 5 years, and from a retrospective study of 1020 patients followed for 10 years [Durante C et al J Clin Endocrinol Metab 2013] reporting that all structural recurrences occurred within 8 years, with 77% occurring within 5 years.

- Percent of patients at 1, 3, and 5 years after randomization with structural incomplete response in the neck defined by the presence of structural or functional evidence of disease, with any Tg level, with or without anti-Tg antibodies
- Percent of patients at 1, 3, and 5 years after randomization with biological incomplete response defined by negative imaging and suppressed Tg ≥1 ng/mL or stimulated Tg≥10ng/mL or rising anti-Tg antibody levels
- Percent of patients at 1, 3, and 5 years after randomization with an indeterminate response defined by nonspecific findings on imaging studies and/or faint uptake in thyroid bed on RAI scanning (if performed) and/or on stimulated Tg detectable but <1ng/mL and/or stable or declining TgAb in the absence of structural or functional disease
- Percent of patients at 1, 3, and 5 years after randomization with diagnosis of distant metastases on metabolic imaging (^131^I, ^18^FDG-TEP) or cross-sectional imaging, and confirmed cytologically (except for metastases with ^131^I uptake) or with repeat imaging at 6 months (if cytology not possible).
- Percent of patients at 1, 3, and 5 years after randomization having undergone further treatment (surgery or 2nd therapeutic administration of 131I, number of retreatments per patient and indication for each retreatment)

#### Functional secondary criteria and quality of life

- At 1 year after randomization (8+/−2 months after 131I administration): percent of patients with persistent hypoparathyroidism and/or persistent vocal fold paralysis; subjective dysphonia (Voice Handicap Index, VHI) and dysphagia (SWAL-QOL) (questionnaires in their validated French versions) compared between groups

The Voice Handicap Index is composed of 10 self-administered questions relating to the functional, physical, and emotional aspects of voice. A French version has been validated [45].

The SWAL-QOL questionnaire is composed of 44 questions concerning eating difficulty, eating duration, eating desire, food selection, fear, and social impact. The validated French version requires approximately 20 min to complete [46].

- At 1, 3, and 5 years after randomization: Quality of life (SF36 + EuroQol EQ-5D), Anxiety (State-Trait Anxiety Inventory-STAI) [47–49].

The SF-36 is the short-form health survey with 36 questions. It yields an 8-scale profile: physical functioning, physical condition, bodily pain, general health, vitality, social functioning, emotional status, mental health, and psychometrically based physical and mental health summary measures [47]. It has been shown to be sensitive for evaluating changes in quality of life in thyroid cancer patients [55]. It can be self-administered in 5–10 min with a high degree of acceptability. The version used in the present study is the version which has a recall period of 1 week. This questionnaire has been employed in a previously published randomized controlled trial on thyroid cancer [40, 55].

EuroQol EQ-5D consists of 5 questions (each with 3 levels of responses) and a self-evaluation of health on a visual analog scale (0–100) [48]. It is employed for the calculation of the utility score for the calculation of QALY (quality-adjusted life years) in cost-utility analysis.

Anxiety will be measured using the Spielberger STAI questionnaire [49]. The STAI state is an instrument for measurement of anxiety. It has 20 questions with four possible responses to each question. Higher scores correspond to higher levels of anxiety. It is suitable for self-administration.

### Outcomes: Cost-utility analysis

A *cost-utility analysis* will be performed to compare total thyroidectomy to total thyroidectomy with bilateral prophylactic central neck dissection. The protocol used will be similar to the one used in ESTIMABL 1 trial and recently published in the Journal of Clinical Oncology [55]. The hypothesis is that TT alone and TTT + PND may differ in terms of the number of patients in complete remission at 1 year or in the number of patients requiring treatment of complications because of recurrent laryngeal nerve injury or hypoparathyroidism.

The horizon time will depend on the results obtained on the main criteria (1 year). If the study confirms the non-inferiority of TT versus TT + PND in terms of oncologic events, a horizon time of 1 year following the initial surgery will be considered.

Consequence will be expressed in QALYs (quality-adjusted life years). Utility score will be assessed using the EQ-5D questionnaire at baseline, 1, 3, and 5 years. QALY will be calculated by multiplying the length of time between two questionnaires by utility score.

Costs will be evaluated from the French collective perspective. Resources consumed by the patient’s management in each strategy will be collected prospectively at baseline, 1, 3, and 5 years.

#### Data collection

Resource consumption collected will concern the following direct medical costs for:

- Hospitalization for initial surgery, including the time in the operating room and for performing the surgery
- Hospitalizations for management of complications (vocal fold paralysis, hypoparathyroidism)
- Hospitalizations for further treatments (surgery or iodine administration)
- Equipment, consultations, medical or paramedical acts for management of complications
- Other direct or indirect costs that are not expected to differ between strategies will not be collected.

#### Cost valuation

Hospital stays (whatever the cause) will be valued using the French DRGs tariffs. Outpatient care costs will be valued on the basis of pricing used by the French health insurance. Tarifs of the General Nomenclature of Professional acts (NGAP), the Common Classification of Medical Acts (CCAM) will be used. Medication will be valued on the base of pricing applied by the French health insurance.

#### Cost-utility analysis

A cost-utility analysis will be performed. One-way sensitivity analyses will be performed by varying all individual costs, incidence of events, and utility variables. Probabilistic sensitivity analyses will be performed using bootstrap resampling to estimate the uncertainty around the incremental cost-utility ratio.

### Participant timeline

**Fig. 3:**
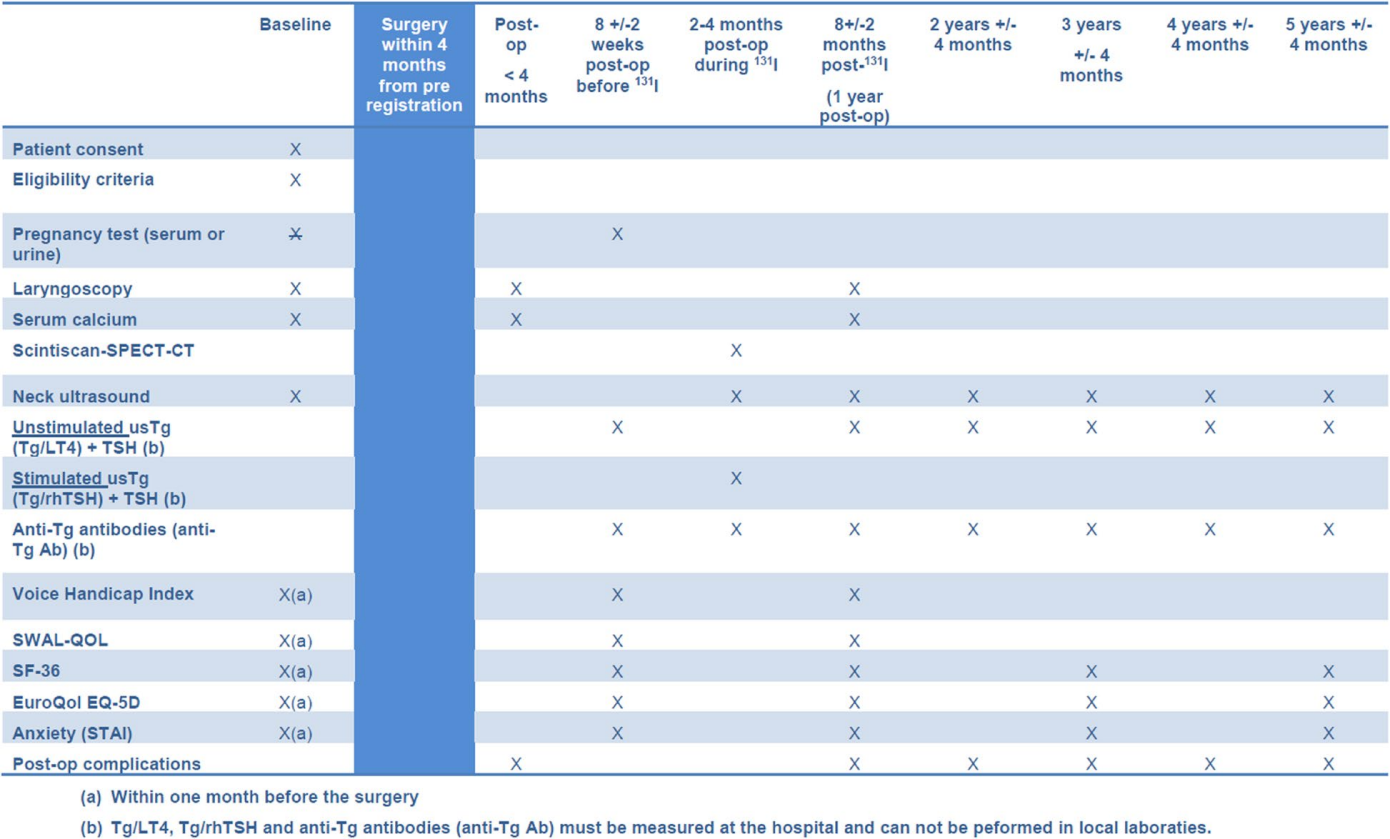
Timeline and criteria for assessments

Figure 3 shows the timeline and criteria for assessments.

### Determination of sample size

Non-inferiority will be demonstrated if the rate of patients in complete remission at 1 year after randomization does not differ by more than ΔL=−5%. Setting the significance level at 0.025 (one-sided) and a power of 80% requires a sample size of 598 patients without Tg antibodies (299 per group) (Nquery software).

In this network’s previous study, 94% of the low-risk patients (including 12% T2N0) were in complete biological remission at 1 year after randomization [40]. In a similar prospective multicenter trial, 90.2% of the low-risk patients, including patients T1-T3 N0-N1 with or without central compartment neck dissection, were in complete biological remission at 1 year [42].

If 5% secondary exclusion (final histopathology not differentiated thyroid cancer): 628 patients are required for randomization.

### Recruitment

Recruiting centers belong to the ENDOCAN-TuThyRef network and are regionally in France high-volume referral centers regularly performing clinical trials on thyroid cancer. This network has already participated in and completed two prospective randomized trials on low-risk differentiated thyroid cancer: the ESTIMABL trial [40] and the ESTIMABL2 trial [41].

## Assignment of interventions: allocation

### Sequence generation

Patients will be prospectively included in the study in 2 steps:

1. Patients will first be registered, after check of eligibility criteria and signature of the informed consent form, before any trial related procedure.
2. Randomization (and validation of the inclusion) will then be performed: (1) before surgery for patients with malignant cytology (Bethesda 6) or (2) in the operating room, after total thyroidectomy and after confirmation of malignancy by intra-operative frozen section analysis for patients with suspicious cytology (Bethesda 5). Randomization will be performed online or by fax with the Trial Master program.

Randomization will be performed online or by fax with the Trial Master program. Patients will be randomly allocated to one of the two treatment groups, based on blocks of participants, stratified by tumor size (cT1b vs cT2) and site (to ensure a balance between the treatment groups at each site). The blocking and stratification measures will not be revealed to those enrolling patients or those allocating the interventions.

Once randomized, patients must be treated as defined in the assigned group and cannot change.

Inclusion/registration and randomization will be performed online or by fax, using the TrialMaster program, and electronically centralized by the Biostatistics and Epidemiology Unit at Gustave Roussy. A fax or an internet access in the operating room or at proximity is then mandatory. In case of any kind of problem, the investigator will contact the study data manager.

The data manager email address, phone, and fax number will be notified in the CRF. In case of any emergency or in the absence of the study data manager, an on-duty data manager will available.

### Concealment mechanism

This is an open study. Patients, physicians, and other care-givers are notified of the randomization group to which the patient is allocated.

### Implementation

The principal investigator of each establishment concerned promises to conduct the clinical trial in conformity with the protocol which has been approved by the Ethic Committee and the competent authority.

The principal investigator should not modify any aspect of the protocol without prior written permission from the Sponsor nor without the approval of the proposed modifications by the Ethic Committee and the competent authority.

The Principal Investigator is responsible for:

- Providing the Sponsor with his/her CV as well as that of co-investigators,
- Ensuring co-investigators and other healthcare professionals should be sufficiently qualified by education, training, and experience to perform their tasks,
- Identifying members of his/her team participating in the trial and defining their responsibilities,
- Recruiting patients after receiving the Sponsor’s approval.

Each investigator is responsible for:

- Personally obtaining the informed consent form which has been dated and signed by the participant in the research prior to any specific trial selection procedure,
- Regularly completing the case report form (CRF) for each patient included in the trial and ensuring that the Clinical Research Assistant (CRA) mandated by the Sponsor has direct access to source documents in order to validate information on the CRF,
- Dating, correcting, and signing the corrections on the CRF for each patient included in the trial,
- Accepting regular visits from a CRA and possibly visits from auditors mandated by the Sponsor or inspectors from the regulatory authorities.

All documentation concerning the trial (protocol, consent form, case report form, investigator file, etc.), as well as the original documents (laboratory results, imaging studies, medical consultation reports, clinical examination reports, etc.), is considered confidential and will be kept in a safe place. The Principal Investigator will keep data as well as a list of patient-identifying data for at least 15 years after the end of the study, or more if specified by the local regulation.

## Assignment of interventions: blinding

This is an open trial, due to the surgical nature of the interventional groups. No blinding will be performed. The data will be anonymized so that the data analyst will be blinded as to group allocation.

### Data collection and management

#### Plans for assessment and collection of outcomes

The Biostatistics and Epidemiology unit at Gustave Roussy will implement electronic CRF (eCRF) using adequate software, thus allowing safe online direct data collection. Quality of life questionnaire will be completed using paper questionnaire.

Each user will have personal identifiers (user ID / password), and data access will be strictly limited according to profiles.

For each patient included in the trial, the eCRF will have to be completed by the hospital CRA and signed by the investigator or the person designated by the investigator.

The study can be interrupted or terminated by the sponsor at any time in agreement with the coordinating investigator. Reasons may include, but are not limited to, the following:

- Frequency and/or unexpected severity of the toxicity,
- If any information leads to doubt as to the benefit/risk ratio of the clinical trial
- Recruitment of patients too low,
- Poor quality of the data collected

The Sponsor has the possibility to replace a site at any time. Reasons for replacing a site may include, but are not limited to, the following:

- Slow recruitment
- Poor protocol adherence / serious breach to the protocol
- Inaccurate or incomplete data recording
- Non-compliance with the International Conference on Harmonisation (ICH) guideline for Good Clinical Practice.

The Sponsor can temporarily or permanently discontinue an investigator for participation in the clinical trial at any time. Reasons may include, but are not limited to, the following:

- Poor protocol adherence / serious breach to the protocol
- Major deviation from the protocol
- Non-compliance with the International Conference on Harmonisation (ICH) guideline for Good Clinical Practice.

Serious breach is defined as any conditions, practices, or processes that adversely affect the rights, safety, or well-being of the subjects and/or the quality and integrity of data.

Major deviation is defined as any conditions, practices, or processes that might adversely affect the rights, safety, or well-being of the subjects and/or the quality and integrity of data.

Minor deviation is defined as any conditions, practices, or processes that would not be expected to adversely affect the rights, safety, or well-being of the subjects and/or the quality and integrity of data.

#### Plans to promote participant retention and complete follow-up

The investigator is responsible for the appropriate medical follow-up of patients until resolution or stabilization of the adverse event or until the patient’s death. This may mean that follow-up should continue once the patient has left the trial.

Follow-up information about a previously reported serious adverse event must be reported by the investigator to the Pharmacovigilance Unit within 24 h of receiving it (on the serious adverse event report form, by ticking the box marked Follow-up N°…). The investigator also transmits the final report at the time of resolution or stabilization of the SAE.

The investigator retains the documents concerning the supposed adverse event so that previously transmitted information can be completed if necessary.

#### Data management

The eCRF will gather both clinical data and quality of life questionnaire (EQ-5D, Voice Handicap Index, SWAL-QOL, SF 36, and STAI) data.

Data collected will be managed in the Biostatistics and Epidemiology unit at Gustave Roussy.

The data collected through the eCRF will be the source data for the analysis. All the statistical analyses will be performed at the Biostatistics and Epidemiology Unit of Gustave Roussy. Therefore, no data transfer will be needed.

In order to guarantee the authenticity and the credibility of the data in conformity with good clinical practices, the Sponsor has installed a quality assurance system which includes:

- Trial management in accordance with the procedures at Gustave Roussy,
- Quality control of data at the investigating site by the Clinical Research Assistant (CRA) in accordance with the monitoring plan,
- Possible auditing of investigating centres.

#### Confidentiality

The investigator promises, on his/her behalf as well as that of all the persons involved in the conduct of the trial, to guarantee the confidentiality of all the information provided by Gustave Roussy until the publication of the results of the trial.

All publications, abstracts, or presentations including the results of the trial require prior approval of the Sponsor (Gustave Roussy).

All oral presentations, manuscripts must include a rubric mentioning the Sponsor, the investigators / institutions that participated in the trial, the cooperative groups, learned societies which contributed to the conduct of the trial, and the bodies which funded the research.

The Study Investigator-Coordinator will write an article reporting on the results as soon as possible after the final analysis and will be the first author of the publication.

The principal investigator will specify the other authors (other investigators, statistician…) in conformity with “Uniform requirements for manuscripts submitted to biomedical journal” (http://www.icmje.org/).

#### Plans for collection, laboratory evaluation, and storage of biological specimens for genetic or molecular analysis in this trial/future use

Each participating center will retain pathology specimens as per French laws, health authorities’ biobanking decrees and routine standard of care, which may be accessed for future studies.

### Statistical methods

#### Statistical methods for primary and secondary outcomes

Descriptive summary statistics will be provided for continuous demographic, laboratory, and clinical variables. The descriptive summary statistics will include number of patients, means and standard deviations for quantitative variables, and percentages for qualitative data.

Subject demographic and baseline characteristics will be summarized by treatment group. The Student test will be used for the continuous variables (or non-parametric test if variables are not normally distributed), and the chi-square test will be used for the categorical variables.

Primary outcomes s1

The main endpoint will be analyzed 1 year after randomization of the last patient, once all the CRF will have been collected and the database has been cleaned. The analysis will be performed when all patients will have 1 year of follow-up, and no lost of follow-up will be tolerated.

A patient will be in remission if the requirements are met at 1 year following randomization. Conversely, a patient will be considered to not be in remission if the criteria are not met at 1 year following randomization. In the absence of censored data, the proportion of patients in complete remission will be calculated as a percentage.

Since the study is designed as a non-inferiority study, the primary analysis will be carried out by considering all evaluable patients (per-protocol population), as this is the most conservative approach in this context. Patient will be considered as evaluable if the treatment and the follow-up conform to the study protocol (diagnostic tests performed) and if the patient does not have detectable anti-Tg antibodies. A sensitivity analysis using the intent-to-treat (ITT) population, considering all patients in their initial group of randomization, will also be performed, to test the robustness of the results.

The observed difference in patients in complete remission (∆) and its 95% unilateral confidence interval will be calculated. If the unilateral confidence interval does not include the −5% clinically relevant difference (∆L), then the TT alone strategy will be considered as non-inferior to the TT + PCND strategy.

All statistical analyses will be performed using the SAS® software.

Secondary outcomes s1

- Unstimulated thyroglobulin levels between groups will be compared between groups using a chi-square test.
- The rate of patients in complete remission at 3 and 5 years will be compared between groups using a chi-square test.
- The rate of patients in with structural incomplete response, biological incomplete response, indeterminate response, distant metastases, or further treatments at 1, 3, and 5 years will be compared between the 2 groups using a chi-square test for each type of response.

For these secondary criteria we expect, based on retrospective series comparing the two surgical techniques (total thyroidectomy alone or total thyroidectomy + PND), rates of incomplete response or re-treatement in the range of the following:

- Difference in locoregional control = 6.9% at 10 years [1]
- Difference in rate of recurrence = 4.6% at 3 years [2]
- Difference in rate of retreatment = 8.2% at 5 years [3]
- Rate of structural recurrence = 1.4% at 8 years [44]
- The rate of patients at 1 year with persistent hypoparathyroidism requiring medication and/or with persistent vocal fold paralysis will be compared between groups using a chi-square test; subjective dysphonia (Voice Handicap Index) and subjective dysphagia (SWAL-QOL) toxicities will be compared using a Student test for each time of evaluation (or a Kruskall-Wallis non-parametric test if they are not normally distributed).
- Quality of life and anxiety: The investigator will inform the patient on the objective of QoL data collection. QoL data may be not exploitable in case of great number of missing questionnaires. Data will be analyzed according to the scoring manual of each questionnaire. A longitudinal analysis using a mixed model will be used to take into account of repeated QoL assessment and the initial value. If this analysis shows a significantly different effect between groups or an interaction between treatment and time, an analysis of the treatment effect on quality of life will be carried out at each time. Mean sub-scale scores will be compared using a Student test for each time of evaluation (or a Kruskall-Wallis non-parametric test if they are not normally distributed).

### Health Economics Analysis

A *cost-utility analysis* will be performed to compare total thyroidectomy to total thyroidectomy with bilateral prophylactic central neck dissection. The protocol used will be similar to the one used in ESTIMABL 1 trial and recently published in the Journal of Clinical Oncology [Borget I et al 2015]. The hypothesis is that TT alone and TTT + PND may differ in terms of the number of patients in complete remission at 1 year or in the number of patients requiring treatement of complications because of recurrent laryngeal nerve injury or hypoparathyroidism.

The horizon time will depend on the results obtained on the main criteria (1 year). If the study confirms the non-inferiority of TT versus TT + PND in terms of oncologic events, a horizon time of 1 year following the initial surgery will be considered.

Consequence will be expressed in QALYs (quality-adjusted life years). Utility score will be assessed using the EQ-5D questionnaire at baseline, 1, 3, and 5 years. QALY will be calculated by multiplying the length of time between two questionnaires by utility score.

Costs will be evaluated from the French collective perspective. Resources consumed by the patient’s management in each strategy will be collected prospectively at baseline, 1, 3, and 5 years.

#### Data collection

Resource consumption collected will concern the following direct medical costs for :

- Hospitalization for initial surgery, including the time in the operating room and for performing the surgery
- Hospitalization for the management of complications
- Hospitalization for further treatments (surgery, radioactive iodine)
- Equipment, consultations, medical or paramedical acts for management of complications
- Other direct or indirect costs that are not expected to differ between strategies will not be collected.

#### Cost valuation

Hospital stays (whatever the cause) will be valued using the French DRGs tariffs. Outpatient care costs will be valued on the basis of pricing used by the French health insurance. Tarifs of the General Nomenclature of Professional acts (NGAP), the Common Classification of Medical Acts (CCAM) will be used. Medication will be valued on the base of pricing applied by the French health insurance.

#### Cost-utility analysis

A cost-utility analysis will be performed. One-way sensitivity analyses will be performed by varying all individual costs, incidence of events, and utility variables. Probabilistic sensitivity analyses will be performed using bootstrap resampling to estimate the uncertainty around the incremental cost-utility ratio.

### Interim analyses

No interim analysis has been planned for this study.

### Methods for additional analyses (e.g., subgroup analyses)

No subgroup analysis has been planned.

### Methods in analysis to handle protocol non-adherence and any statistical methods to handle missing data

Since the study is designed as a non-inferiority study, the primary analysis will be carried out by considering all evaluable patients (per-protocol population), as this is the most conservative approach in this context. A patient will be considered as evaluable if the treatment and the follow-up conform to the study protocol (diagnostic tests performed) and if the patient does not have detectable anti-Tg antibodies. A sensitivity analysis using the intent-to-treat (ITT) population, considering all patients in their initial group of randomization, will also be performed, to test the robustness of the results.

### Plans to give access to the full protocol, participant-level data, and statistical code

Public access of the protocol, data, and statistical code will be available at the end of the trial and after publication by contacting the principal investigator.

### Oversight and monitoring

Monitoring will be performed regularly in all participating centers, with oversight performed by the promotor.

#### Composition of the coordinating center and trial steering committee

The coordinating center is located at the promotor’s site. It is composed of the principal investigator, the methodologist and statistician, the promotor data manager, and the promotor study coordinator. They will meet annually and in addition whenever there are queries from participating centers. In each participating center, the site investigator and study coordinator will meet annually and in case of queries. The promotor’s clinical research committee will adjucate the end of the trial.

#### Composition of the data monitoring committee, its role and reporting structure

The promotor’s data management and monitoring team is composed of twelve data managers and their assistants employed exclusively for clinical studies. One data manager is assigned to the present study with backup from the team. The data management team at the promotor site is certified by the French National Cancer Institut (“Centre de Traitement de Données CTD, Institut National du Cancer”). This data management team is linked to the promotor and is independent from the national institution financing the study.

### Adverse event reporting and harms

An adverse event (AE) is any untoward medical occurrence in a patient that does not necessarily have a causal relationship with the study intervention/procedure (thyroidectomy, neck dissection, radioiodine, and rhTSH administration). An AE can therefore be any unfavorable or unintended sign (including an abnormal laboratory finding), symptom, or disease temporarily associated with a trial procedure.

A serious adverse event (SAE) is any untoward medical occurrence that at any dose:

- Is fatal (results in death)
- Is life-threatening
- Requires or prolongs inpatient hospitalization*
- Results in persistent or significant disability / incapacity
- Is a congenital anomaly / birth defect
- Is medically significant**

* Hospitalization is defined as an unplanned, formal inpatient admission, even if the hospitalization is a precautionary measure for continued observation. Thus hospitalization for protocol treatment, elective procedures (unless brought forward due to worsening symptoms), or social reasons are not regarded as a SAE.

** Medical judgment should be exercised in deciding whether an AE is serious in other situations. AEs that are not immediately life-threatening or do not result in death or hospitalization but may jeopardize the subject or may require intervention to prevent one of the other outcomes listed in the SAE definition above, should be considered serious.

Events exclusively related to tumor relapse / progression or treatment of tumor relapse / progressions are not considered as SAE.

Adverse events associated with surgery (thyroidectomy, neck dissection) are as follows: hematoma, postoperative bleeding, paralysis of the vocal cord, speech disorders, voice change, swallowing disorders, breathing disorders, hypocalcaemia, lymphatic leakage, wound infection, and nerve damage other than the recurrent nerve.

## The following are not considered to be serious adverse events (SAE):

- A visit to the emergency room or other hospital department for less than 24 h that does not result in admission (unless considered an “important medical event” or a life-threatening event)
- Outpatient or same-day or ambulatory procedures
- Observation or short-stay units
- Hospitalization due to diagnostic procedures or standard supportive care (e.g., implant of central venous catheter)
- A pre-planned hospitalization for a condition which existed at the start of study drug and which did not worsen during the course of study drug treatment
- Social admission (e.g., subject has no place to sleep; hospice facilities)
- Administrative admission (e.g., for yearly physical examinations)
- Protocol-specified admission during a clinical trial (e.g., for a procedure required by the study protocol or for clinical research)
- Optional admission not associated with a precipitating clinical AE (e.g., for elective cosmetic surgery)

All adverse events will be evaluated and graded according to the Clavien-Dindo classification of surgical complications (Annex 7). Any SAE which occurs or comes to the attention of the investigator at any time during the study since consent is given and within 30 days after the last study procedure, independent of the circumstances or suspected cause, must be reported immediately, within 24 h of knowledge (at latest on the next working day) by fax via a SAE report form to the Pharmacovigilance Unit at IGR.

All late serious adverse events (occurring after this period of 30 days) considered to be reasonably related to the study treatment(s) or the research must be declared (no time limit).

Information collected in the SAE form is crucial to assess the case. For this reason, diligence in collecting as much verifiable and reliable information is needed: both quality and timeliness are key factors. If known, the diagnosis of the underlying illness or disorder should be recorded, rather than its individual symptoms. The following information should be captured for all SAEs: onset, duration, intensity, seriousness, relationship to study procedure, action taken, and treatment required.

The investigator must also attach the following to the serious adverse event report form, wherever possible:

- A copy of the summary of hospitalization or prolongation of hospitalization
- A copy of the post-mortem report (if applicable)
- A copy of all laboratory examinations and the dates on which these examinations were carried out, including relevant negative results, as well as normal laboratory ranges.
- All other document that he judges useful and relevant.

All these documents will remain anonymous.

Further information can be requested (by fax, telephone or when visiting) by the monitor and/or the safety manager.

The Pharmacovigilance Unit at IGR will assess the SAE in terms of seriousness, severity (Clavien-Dindo), relationship to the study procedure, and expectedness. All SAEs will be coded using MedDRA.

To comply with regulatory requirements, the sponsor will identify and report all SAEs that are related to the study procedures and unexpected (i.e., not described in the protocol). In the European Union, an event meeting these criteria is termed as suspected unexpected serious adverse reaction (SUSAR).

### Frequency and plans for auditing trial conduct

The pharmacovigilance unit at Gustave Roussy will issue once a year throughout the clinical trial, or on request, the annual safety report (ASR) of the study. Annual audits will be conducted by the promotor.

### Plans for communicating important protocol amendments to relevant parties (e.g., trial participants, ethical committees)

Each amendment will be subjected to a national ethics committee (CPP) for approval. Amendments will be communicated directly by the promotor to the participating centers who will, if applicable, inform trial participants.

### Dissemination plans

The trial results will be published by the promotor team in a peer-reviewed medical journal. The results will be proposed for podium presentations in international specialized congresses. There are no legal restrictions to these types of communication.

Each participating patients will be informed of the results of the trial when completed.

## Discussion

The main objective of this study is to show the non-inferiority of total thyroidectomy alone as compared to a total thyroidectomy with a bilateral prophylactic central neck dissection in clinically low-risk patients. This trial will ultimately include a number of patients with intermediate risk factors (Haugen) found on finally pathology. This is the rationale for administering an ablative dose of RAI to all patients, despite the fact that RAI is no longer systematically indicated for pathologically low-risk tumors.(Leboulleux 2022). A secondary objective, however, is to also compare the thyroglobulin levels 8 weeks after surgery and before administration of RAI in each group, to evaluate outcomes without RAI. The need for a total thyroidectomy for low-risk tumors has also been questioned (Haugen); unfortunately, this clinical trial is designed only to answer one question, that is, the utility of PND, and is not suited to answer other questions concerning “downgrading” of surgery for low-risk tumors.

Centers not including patients may be subjected to closing. Centers in France and outside of France may be added as investigators, subjected to approval of an amendment to the study by the national authorities (National Agency for Drug Safety, “Agence Nationale de la Sécurité du Médicament et des Produits de Santé, ANSM) and the French nationa research ethics committee (Comité de Protection des Personnes, CPP). In the event of other amendments, they will also be subjected to the above-cited national authorities for safety and ethical evaluation. The promotor data managing committee will communicate any approved protocol modifications to the participating centers.

## Trial status

Accrural began on August 29, 2018. The current protocol is version 2.3, October 22, 2021.The estimated completion date for the primary endpoint is June 2026.

Accrural will be carried out for 6 years, for a total duration of 11 years for the study (5 years after randomization of the last patient).

## Supplementary Information

**Additional file 1: Annex 1.**

**Additional file 2: Annex 2.**

**Additional file 3: Annex 3.**

**Additional file 4: Annex 4.**

**Additional file 5: Annex 5.**

**Additional file 6: Annex 6.**

**Additional file 7: Annex 7.**

**Additional file 8: Annex 8.**

**Additional file 9: Annex 9.**

**Additional file 10: Annex 10.**

## Data Availability

Data collected will be managed in the Biostatistics and Epidemiology unit at Gustave Roussy. The data collected through the eCRF will be the source data for the analysis. All the statistical analyses will be performed at the Biostatistics and Epidemiology Unit of Gustave Roussy.

## Abbreviations

TT: Total thyroidectomy
PND: Prophylactic neck dissection
RAI, ^131^I: Radioactive iodine
FNAB: Fine-needle aspiration biopsy
SPECT-CT: Single-photon emission computed tomography combined with computed tomography
mCi: Millicuries
GBq: Giga becquerels
TSH: Thyroid-stimulating hormone
rhTSH: Recombinant human thyroid-stimulating hormone (injectable)
Tg: Thyroglobulin
LT4: L-thyroxine therapy
usTg/LT4: Ultrasensitive thyroglobulin measured during L-Thyroxine treatment
rhTSH/Tg: Stimulated thyroglobulin assay
TgAb, anti-Tg Ab: Serum anti-thyroglobulin antibodies
US: Ultrasound
QoL: Quality of life
VHI: Voice Handicap Index
SWAL-QOL: Swallowing quality of life questionnaire
SF36: Quality of life questionnaire, short-form 36
EQ-5D: EuroQuol quality of life visual analog scale
STAI: State-Trait Anxiety Inventory
QALY: Quality-adjusted life years
ß-HCG: Beta-human chorionic gonadotropin test (pregnancy test)
