# Correction: ESTIMation of the ABiLity of prophylactic central compartment neck dissection to modify outcomes in low-risk differentiated thyroid cancer: a prospective randomized trial

**DOI:** 10.1186/s13063-023-07467-x

**Published:** 2023-07-11

**Authors:** Dana Hartl, Yann Godbert, Xavier Carrat, Stéphane Bardet, Audrey Lasne-Cardon, Pierre Vera, Elena Ilies, Slimane Zerdoud, Jérôme Sarini, Mohamad Zalzali, Luigi La Manna, Olivier Schneegans, Antony Kelly, Philppe Kaufmann, Patrice Rodien, Laurent Brunaud, Solange Grunenwald, Elie Housseau, Salim Laghouati, Nathalie Bouvet, Elodie Lecerf, Julien Hadoux, Livia Lamartina, Martin Schlumberger, Isabelle Borget

**Affiliations:** 1 Gustave Roussy, 114 rue Edouard Vaillant, 94805 Villejuif Cedex, France; 2 Institut Bergonié, 229 Cr de l’Argonne, 33076 Bordeaux Cedex, France; 3 Centre François Baclesse, 3 Av. du Général Harris, 14000 Caen, France; 4 Centre Henri Becquerel, 1 Rue d’Amiens, 76038 Rouen, France; 5 Institut Universitaire du Cancer de Toulouse, 1 avenue Irène Joliot-Curie, 31059 Toulouse Cedex 9, France; 6 Institut Godinot, 1 Rue du Général Koenig, 51100 Reims, France; 7 ICANS Institut de Cancérologie Strasbourg Europe, 17 Rue Albert Calmette, 67200 Strasbourg, France; 8 Centre Jean Perrin, 58, rue Montalembert, 63011 Clermont-Ferrand Cedex 01, France; 9 Centre Hospitalier Universitaire d’Angers, 4 Rue Larrey, 49100 Angers, France; 10 Centre Hospitalier Régional et Universitaire de Nancy, Rue du Morvan, 54000 Nancy, France; 11 Centre Hospitalier Universitaire de Toulouse, 2 Rue Charles Viguerie, 31300 Toulouse, France; 12 Centre Hospitalier de Hagenau, 64 Av. du Professeur René Leriche, 67500 Haguenau, France

**Correction: BMC Trials 24, 298 (2023)**

**https://doi.org/10.1186/s13063-023-07294-0**

Following publication of the original article [1], we have been informed that the author affiliations have been incorrectly assigned. Originally, all authors were linked to Gustave Roussy, 114 rue Edouard Vaillant, 94805, Villejuif Cedex, France.

The original article has been corrected.
