# Supplementary material for: ESTIMation of the ABiLity of prophylactic central compartment neck dissection to modify outcomes in low-risk differentiated thyroid cancer: a prospective randomized trial
Source: Trials. 2023 Apr 28;24:298. doi: 10.1186/s13063-023-07294-0 (PMC10142499; doi:10.1186/s13063-023-07294-0)
Supplement: Supplementary file 1 — Additional file 1: Annex 1. [file 13063_2023_7294_MOESM1_ESM.pdf]

NOTE D'INFORMATION & FORMULAIRE DE CONSENTEMENT

"Estimation de l'impact de l'évidement ganglionnaire prophylactique du compartiment central du cou sur les résultats oncologiques des cancers différenciés de la thyroïde à bas risque de récidive loco-régionale"

"ESTIMABL3"

N°CSET 2017/2581
N° d'enregistrement 2017-A01779-44

(Original pour le médecin-investigateur, copie à remettre au patient)

Investigateur Coordonnateur : Dr HARTL

Promoteur : Gustave Roussy

Gustave Roussy promoteur

GUSTAVE ROUSSY
CANCER CAMPUS
GRAND PARIS

Madame, Monsieur,

Le médecin vous propose de participer à une étude clinique. Cette recherche est à l'initiative de Gustave Roussy qui est promoteur de cette étude. Le médecin qui vous suivra pendant l'étude est appelé médecin-investigateur.

Le médecin-investigateur vous a déjà informé(e) oralement des modalités de cette recherche. Ce formulaire a pour objet de vous fournir un résumé concernant les différents aspects de l'étude, les éventuels bénéfices, risques et gênes qu'elle pourrait occasionner.

Nous vous proposons de lire attentivement cette note d'information qui essaye de répondre aux questions que vous êtes susceptible de vous poser avant de décider si vous souhaitez ou non participer à cette étude. Prenez le temps de bien lire ces informations et n'hésitez pas à poser des questions au médecin-investigateur si vous souhaitez avoir des précisions sur certains points. Vous pouvez également en discuter avec votre entourage ou votre médecin traitant avant de prendre votre décision.

Vous pourrez à tout moment durant l'étude vous adresser au médecin-investigateur pour lui poser des questions complémentaires.

/// LES OBJECTIFS DE LA RECHERCHE

Votre médecin vous propose de participer à cette étude car vous allez être opéré(e) :

- pour un cancer de la thyroïde avec une *thyroïdectomie totale**
- ou bien pour un *nodule thyroïdien** qui est suspect de cancer

En l'absence de ganglion suspect à l'échographie, l'utilité d'une chirurgie des ganglions pour les cancers de la thyroïde reste controversée. Il n'est pas certain que cette pratique apporte un bénéfice en termes de guérison d'une maladie qui a déjà un taux de guérison élevé. C'est pourquoi, il existe actuellement deux approches en France :

- ✓ une chirurgie systématique des ganglions, préconisée par la Société Française d'Oto-Rhino-Laryngologie et de Chirurgie de la Face et du Cou (SFORL)
- ✓ alors que l'Association Francophone de Chirurgie Endocrinienne (AFCE) ne la préconise pas de manière systématique

Notre hypothèse est qu'il n'y a pas de différence entre les deux approches mais nous souhaitons les comparer dans le cadre d'une étude scientifique pour savoir quelle pratique sera à privilégier dans l'avenir.

Les objectifs secondaires de cette étude sont de comparer les deux approches en termes de toxicité, de qualité de vie. Nous réaliserons une étude médico-économique.

/// LE TRAITEMENT DE L'ETUDE

- Si vous avez un cancer de la thyroïde, votre dossier a déjà été présenté en réunion de concertation pluridisciplinaire (RCP) qui a confirmé que vous deviez être opéré(e).

- Si vous avez un nodule thyroïdien qui est suspect de cancer, un examen *extemporané** du nodule devra être réalisé pendant l'opération, pour confirmer le diagnostic et valider votre participation dans l'étude.

Si vous acceptez de participer à cette étude, votre chirurgien effectuera la thyroïdectomie totale comme réalisée en pratique courante. Puis, - à condition que le diagnostic de cancer soit confirmé par l'examen extemporané en cas de nodule suspect – il procédera à un tirage

au sort : soit il réalisera une chirurgie des ganglions, soit il ne la réalisera pas. C'est ce que l'on appelle « randomisation ».
50% des patients inclus dans l'étude auront une chirurgie des ganglions et 50% des patients auront une chirurgie sans chirurgie des ganglions.
En complément de la chirurgie, le traitement habituel par iode radioactif sera maintenu après la chirurgie.

/// LE DEROULEMENT DE L'ETUDE

Si vous acceptez de participer à cette étude, vous devrez signer le formulaire de consentement joint à ce document.

Le déroulement des examens avant l'opération, de l'hospitalisation et du suivi sera le même que si vous ne participiez pas à l'étude et quel que soit le résultat du tirage au sort au bloc opératoire.

Avant la chirurgie, vous serez vu(e) en consultation par votre chirurgien et par le médecin anesthésiste. Votre chirurgien vous expliquera les risques de l'intervention, qui sont les mêmes quel que soit le résultat du tirage au sort au bloc opératoire.

Une partie de la tumeur prélevée au moment de la chirurgie sera récupérée et envoyée dans un laboratoire (module HistoCytoPathologie) à Gustave Roussy pour réaliser des analyses complémentaires, notamment sur les mutations qui pourraient être présentes au niveau de la tumeur. Aucun prélèvement supplémentaire ne sera nécessaire.

Après l'opération vous serez revu(e) en consultation par votre chirurgien et par un médecin endocrinologue ou spécialiste de la médecine nucléaire qui vous expliquera le déroulement du traitement par l'iode radioactif. Ce traitement est le traitement standard des patients atteints de la même maladie que vous ; il vous aurait été administré que vous participiez ou non à cette étude. Ce traitement se fera dans les 4 mois qui suivent votre chirurgie.

Après ce traitement, vous serez revu(e) environ 8 mois plus tard, puis tous les ans, avec une prise de sang et une échographie du cou pendant 5 ans. A chaque fois, le dosage des hormones de la thyroïde (tyroglobuline et anticorps anti-Tg) devra être réalisé à l'hôpital.

On vous demandera également de remplir des questionnaires concernant votre qualité de vie avant la chirurgie, puis 1 an, 3 et 5 ans après l'opération (environ 80 questions).

/// TABLEAU RECAPITULATIF DES VISITES

	Avant l'opération	Chirurgie	8 +/- 2 semaines après la chirurgie AVANT l'iode	2 à 4 mois après la chirurgie PENDANT l'iode	Un an après la randomisation	Tous les ans pendant 5 ans
**Test de grossesse**			X			
**Examen du larynx**	X				X	
**Prise de sang (à l'hôpital)**	X		X	X	X	X
**Scintigraphie***				X		
**Échographie du cou**	X			X	X	X
**Questionnaires sur la voix et la déglutition**	X		X		X	A 3 et 5 ans
**Questionnaires sur la qualité de vie et l'anxiété**	X		X		X	A 3 et 5 ans

/// NOMBRE DE PARTICIPANTS ET DUREE DE L'ETUDE

Il est prévu d'inclure environ 1000 patients dans cette étude en France.

/// LES BENEFICES ATTENDUS

Le traitement chirurgical et le traitement par l'iode radioactif sont des traitements standards, quel que soit le résultat du tirage au sort au bloc opératoire. Les deux approches – chirurgie systématique ou non des ganglions - sont des traitements acceptés par les sociétés savantes en France et dans le monde.

Le résultat de cette étude pourra permettre de ne plus faire une chirurgie systématique des ganglions pour les patients à l'avenir.

/// LES RISQUES

➔ Les risques de la chirurgie sont les mêmes, quel que soit le résultat du tirage au sort au bloc opératoire (chirurgie ou non des ganglions) :
- Un hématome (saignement sous la peau) peut survenir dans les heures ou pendant la nuit qui suivent l'intervention et peut nécessiter une réintervention au bloc opératoire.
- Un abcès (une infection locale) peut survenir, généralement plusieurs jours après l'intervention, et peut nécessiter *un drainage** en consultation et/ou un traitement par antibiotiques.
- Une paralysie d'une corde vocale peut survenir de manière imprévisible, malgré une technique minutieuse. Cette paralysie se manifeste par une modification de la voix qui devient plus rauque et plus faible, et peut parfois s'accompagner d'une difficulté respiratoire à l'effort. Des difficultés à avaler, notamment des fausses-routes à l'ingestion des liquides, peuvent survenir dans les premiers jours, mais s'estompent généralement en quelques jours ou semaines. La voix récupère spontanément pour la majorité des patients en 1

à 6 mois. La paralysie définitive est rare, et si les troubles de la voix persistent, ils peuvent être améliorés par une chirurgie de la corde vocale.

- Une baisse du taux de calcium dans le sang : le calcium sera dosé par une prise de sang après l'opération, et un traitement préventif par des comprimés de calcium et de la vitamine D est souvent prescrit. Une baisse du calcium est généralement transitoire, mais peut nécessiter des suppléments de calcium pendant quelques semaines ou quelques mois.

  Quelques études ont trouvé une légère augmentation de l'hypoparathyroïdie (production insuffisante d'hormone par les glandes parathyroïdes) transitoire après chirurgie des ganglions versus sans chirurgie des ganglions, mais d'autres études ne signalent pas ce problème.
  Les risques de séquelles définitives sont les mêmes (avec ou sans chirurgie des ganglions).

  Après une thyroïdectomie totale, un traitement pour remplacer les hormones produites par la thyroïde est systématiquement administré.

➔ Les risques du traitement par l'iode radioactif (administré à tous les patients dans l'étude quel que soit le résultat du tirage au sort) :

- Nausées de courte durée suite aux injections intramusculaires de TSH
- Nausées lors de la prise de la gélule d'iode
- Perte transitoire du goût (quelques jours)
- Sensations désagréables dans les glandes salivaires (quelques jours)

/// CONTRACEPTION ET GROSSESSE

Une intervention chirurgicale sous anesthésie générale pendant la grossesse peut mettre en péril la santé voire la vie de l'enfant à naître. Il est nécessaire de prévenir votre chirurgien si vous êtes enceinte pour décaler la date de l'opération pour une date après l'accouchement.
Le traitement par l'iode radioactif est formellement contre-indiqué pendant la grossesse.
Une contraception efficace est nécessaire pendant toute la durée de l'étude et pendant les 6 mois qui suivent tout traitement par l'iode radioactif pour éviter une grossesse pendant cette période.

/// DEVENIR DES ECHANTILLONS ET DES DONNEES

L'utilisation ultérieure de vos échantillons biologiques dans un autre cadre que celui de cette étude n'est possible que si vous y avez consenti. Si vous l'acceptez, vos échantillons biologiques seront conservés après la fin de l'étude dans une structure agréée par le ministère de la Recherche, et pourront être utilisés à des fins de recherche scientifique et médicale sur votre maladie. Une partie des prélèvements biologiques pourra être transférée dans le cadre d'un partenariat de recherche à d'autres institutions/organismes universitaires et partenaires industriels en accord avec les dispositions du Code de la santé publique. Votre décision est révocable à tout moment et vous pourrez vous opposer à cette utilisation en le signifiant au médecin investigateur.

De même, l'utilisation ultérieure des données collectées dans le cadre de futures recherches scientifiques, en dehors de ce protocole de recherche clinique n'est possible que suite à l'accord du Comité de Protection des Personnes et si vous y consentez. Vous conservez à tout moment votre droit d'accès, d'opposition et de rectification conformément au droit applicable relatif à la protection des données.

/// LES ALTERNATIVES THERAPEUTIQUES

Le médecin-investigateur pourra vous donner davantage d'informations à propos des risques et bénéfices liés à votre participation à cette étude clinique par rapport à d'autres alternatives thérapeutiques. N'hésitez pas à lui poser la question avant de prendre votre décision.
Dans tous les cas, vous bénéficierez d'une prise en charge appropriée.

/// PARTICIPATION VOLONTAIRE / INTERRUPTION DE PARTICIPATION

Votre participation à cette étude est entièrement libre et volontaire. Vous devez être affilié(e) à un régime de sécurité sociale ou bénéficiaire d'un tel régime (y compris la CMU) pour pouvoir y participer. Vous ne pouvez pas participer simultanément à une autre étude.

Vous pouvez décider de refuser de participer ou de sortir de l'étude à tout moment, sans avoir à vous justifier et sans que cela n'ait de conséquence sur votre suivi médical ni sur la qualité de vos soins ultérieurs. Nous vous demandons simplement de bien vouloir le préciser au médecin-investigateur.

Si vous retirez votre consentement, les informations et les échantillons recueillis dans le cadre de cette étude seront conservés et analysés, sauf si vous exprimez votre refus auprès du médecin investigateur. Dans ce cas, les données de l'étude et les échantillons vous concernant seront détruits.

/// NOUVELLES INFORMATIONS SUR L'ETUDE ET SUR LE TRAITEMENT A L'ETUDE

Vous serez informé(e) en temps opportun de toute nouvelle information concernant votre sante ou donnée nouvelle qui pourrait influencer votre volonté de poursuivre votre participation à cette étude. Si de nouvelles informations venaient à modifier celles qui vous sont présentées dans ce document, un nouveau document vous serait remis afin que vous en soyez informé(e) et afin que vous confirmiez, ou non, votre souhait de poursuivre cette étude.

/// COÛTS POTENTIELS / INDEMNISATION

Vous ne serez pas payé(e) pour votre participation à cette étude.
Vous bénéficierez gratuitement de tous les examens exclusivement requis pour cette étude, et qui ne font pas partie de vos soins médicaux réguliers.

/// ASSURANCE

Le promoteur de cet essai qui en assure la gestion et la responsabilité est Gustave Roussy – 114 rue Edouard Vaillant – 94 805 VILLEJUIF - France.
Gustave Roussy a souscrit une assurance (n° de contrat 124.895) auprès de la Société SHAM France) dans les conditions prévues à l'article L. 1121-10 du code de la santé publique, garantissant sa responsabilité civile et celle de tout intervenant en cas de conséquences dommageables de la recherche pour la personne qui s'y prête ou pour ses ayants droit. Si vous estimez avoir subi un préjudice du fait de votre participation à cette étude, vous devrez en informer votre médecin-investigateur.

/// PROTECTION DES PATIENTS / CONFIDENTIALITE DES DONNEES

Conformément au Code de la Santé Publique (Titre II) relatif aux recherches impliquant la personne humaine et les Bonnes Pratiques Cliniques, ce protocole a reçu l'avis favorable d'un Comité de Protection des Personnes (CPP IDF 2) le 13/02/2018.

Conformément aux recommandations du Plan Cancer, ce document a été soumis pour relecture, avis et conseil au comité de patients de la Ligue nationale contre le cancer.

En complément, cette étude est menée conformément aux dispositions de l'article 13 du Règlement Européen (UE) 2016/679 relatif à la protection des personnes physiques à l'égard du traitement des données à caractère personnel et à la libre circulation de ces données reproduit ci-après dans son intégralité en Annexe 1.

Tous les fichiers et les dossiers dans lesquels apparaîtra votre identité seront gardés par le médecin-investigateur et resteront strictement confidentiels. Votre dossier médical restera confidentiel et ne pourra être consulté que par les autorités de santé et par des personnes dûment mandatées par le promoteur de l'essai et soumises au secret professionnel, sous la responsabilité de votre médecin. Conformément à la loi n°2002-303 du 4 mars 2002 relative aux droits des malades et à la qualité du système de santé vous avez un droit d'accès à votre dossier médical.

Dans le cadre de cette recherche, un traitement de vos données personnelles va être mis en œuvre pour permettre d'analyser les résultats de l'étude. L'utilisation ultérieure des données collectées dans le cadre de futures recherches scientifiques, en dehors de cette étude, est également possible. L'accord du Comité de Protection des Personnes devra être demandée dans ce cas.

Le traitement se fera de manière anonyme et confidentielle : les données seront identifiées par un numéro de code et/ou vos initiales ou les trois premières lettres de votre nom/prénom.

Ces données seront, dans des conditions assurant leur confidentialité, transmises au promoteur de la recherche, et pourront également être transmises à des personnes ou sociétés agissant pour son compte, à des personnes ou sociétés partenaires ou, le cas échéant, à des autorités de santé françaises ou étrangères. Dans ce cadre, les données pourront être transférées en dehors de la France, y compris vers des pays non-européen. Les destinataires peuvent éventuellement être situés dans un pays ne bénéficiant pas d'un niveau de protection des données équivalent à celui en vigueur au sein de l'Union Européenne. Des clauses contractuelles types sécuriseront le transfert des données en dehors de l'Union européenne afin d'apporter le même niveau de protection des données que l'Union européenne. Cependant, le promoteur prendra toutes les mesures nécessaires à fin d'assurer la protection et la confidentialité des données, comme de garantir votre anonymat.

Le traitement des données est soumis à la loi n° 78-17 du 6 janvier 1978 modifiée, relative à l'informatique, aux fichiers et aux libertés Il est conforme aux requis imposés par la méthodologie de référence, éditée par la CNIL, « MR-001 ». Il a fait l'objet d'une procédure d'autorisation auprès de la CNIL.
Conformément aux dispositions de la loi relative à l'informatique, aux fichiers et aux libertés, précitée, vous disposez d'un droit d'accès et de rectification. Vous disposez également d'un droit d'opposition à la transmission des données couvertes par le secret professionnel susceptibles d'être traitées. Vous pouvez exercer votre droit à tout moment en vous adressant au médecin-investigateur.

Le médecin investigateur pourra être amené à communiquer des informations sur votre état de santé après la fin de l'étude (par exemple votre statut vital, l'évolution de votre maladie ou

l'évolution ou la survenue d'un effet indésirable) si l'utilité de ces données est justifiée aux fins des objectifs de l'étude.

Les résultats de l'étude pourront être communiqués à la communauté scientifique lors de séminaires, de congrès ou publiés dans la presse scientifique, en garantissant la confidentialité absolue des données vous concernant, ainsi que votre anonymat.
Selon les dispositions de l'article L 1122-1 du Code de la Santé Publique, les patients ayant participé à l'étude seront informés, à leur demande, par les médecins-investigateurs des résultats globaux de cette recherche à son terme.

Enfin, nous vous informons que votre médecin traitant sera informé de votre participation à l'étude, sauf si vous exprimez votre refus auprès du médecin investigateur.

/// LEXIQUE

*Thyroidectomie totale: ablation des deux lobes de la thyroïde

*Un nodule thyroïdien est une petite grosseur qui se forme sur la glande thyroïde, située à la base du cou. Cette tuméfaction peut correspondre à des lésions très variées : tumeur bénigne (adénome), kyste, cancer.

*Extemporané : se dit d'un prélèvement fait et examiné pendant le déroulement de l'intervention chirurgicale

*Radioprotection : Ensemble des mesures prises pour assurer la protection de l'homme et de son environnement contre les effets néfastes des rayonnements ionisants.

*La scintigraphie est un examen d'imagerie médicale permettant d'étudier la structure de la glande thyroïde et d'en observer le fonctionnement grâce à l'injection d'un produit faiblement radioactif, qui présente la particularité de se fixer préférentiellement sur les cellules hyperactives de l'organe et d'émettre des signaux (rayons gamma). Ces signaux sont analysés grâce à une gamma-caméra. La répartition du produit est ensuite visualisée à un écran couplé à la gamma-caméra, sous forme de points lumineux, décrivant la structure et le fonctionnement de la thyroïde.

*Le drainage est un acte médical ou chirurgical qui consiste à récolter des fluides produits par l'organisme, et à les évacuer soit vers l'extérieur, soit vers l'intérieur du corps.

/// CONTACTS POUR INFORMATIONS COMPLEMENTAIRES OU EN CAS D'URGENCE

Si vous souhaitez obtenir des informations complémentaires ou si vous rencontrez un problème quelconque au cours de votre participation, veuillez contacter le médecin-investigateur, ou un membre de son équipe équipe, ou le coordonnateur de l'étude, le Dr Dana HARTL, au numéro suivant : 01 42 11 46 12 (Gustave Roussy)

Ou tampon

Nom : …………………………………………………….
Téléphone : …………………………………………….

ANNEXE 1

Article 13 du Règlement Européen (UE) 2016/679 relatif à la protection des personnes physiques à l'égard du traitement des données à caractère personnel et à la libre circulation de ces données

Article 13

Informations à fournir lorsque des données à caractère personnel sont collectées auprès de la personne concernée

1. Lorsque des données à caractère personnel relatives à une personne concernée sont collectées auprès de cette personne, le responsable du traitement lui fournit, au moment où les données en question sont obtenues, toutes les informations suivantes:

a) l'identité et les coordonnées du responsable du traitement et, le cas échéant, du représentant du responsable du traitement

b) le cas échéant, les coordonnées du délégué à la protection des données;

c) les finalités du traitement auquel sont destinées les données à caractère personnel ainsi que la base juridique du traitement;

d) lorsque le traitement est fondé sur l'article 6, paragraphe 1, point f), les intérêts légitimes poursuivis par le responsable du traitement ou par un tiers;

e) les destinataires ou les catégories de destinataires des données à caractère personnel, s'ils existent; et

f) le cas échéant, le fait que le responsable du traitement a l'intention d'effectuer un transfert de données à caractère personnel vers un pays tiers ou à une organisation internationale, et l'existence ou l'absence d'une décision d'adéquation rendue par la Commission ou, dans le cas des transferts visés à l'article 46 ou 47, ou à l'article 49, paragraphe 1, deuxième alinéa, la référence aux garanties appropriées ou adaptées et les moyens d'en obtenir une copie ou l'endroit où elles ont été mises à disposition;

2. En plus des informations visées au paragraphe 1, le responsable du traitement fournit à la personne concernée, au moment où les données à caractère personnel sont obtenues, les informations complémentaires suivantes qui sont nécessaires pour garantir un traitement équitable et transparent:

a) la durée de conservation des données à caractère personnel ou, lorsque ce n'est pas possible, les critères utilisés pour déterminer cette durée;

b) l'existence du droit de demander au responsable du traitement l'accès aux données à caractère personnel, la rectification ou l'effacement de celles-ci, ou une limitation du traitement relatif à la personne concernée, ou du droit de s'opposer au traitement et du droit à la portabilité des données;

c) lorsque le traitement est fondé sur l'article 6, paragraphe 1, point a), ou sur l'article 9, paragraphe 2, point a), l'existence du droit de retirer son consentement à tout moment, sans porter atteinte à la licéité du traitement fondé sur le consentement effectué avant le retrait de celui-ci;

d) le droit d'introduire une réclamation auprès d'une autorité de contrôle;

e) des informations sur la question de savoir si l'exigence de fourniture de données à caractère personnel a un caractère réglementaire ou contractuel ou si elle conditionne la conclusion d'un contrat et si la personne concernée est tenue de fournir les données à caractère personnel, ainsi que sur les conséquences éventuelles de la non-fourniture de ces données;

f) l'existence d'une prise de décision automatisée, y compris un profilage, visée à l'article 22, paragraphes 1 et 4, et, au moins en pareils cas, des informations utiles concernant la logique sous-jacente, ainsi que l'importance et les conséquences prévues de ce traitement pour la personne concernée.

3. Lorsqu'il a l'intention d'effectuer un traitement ultérieur des données à caractère personnel pour une finalité autre que celle pour laquelle les données à caractère personnel ont été collectées, le responsable du traitement fournit au préalable à la personne concernée des informations au sujet de cette autre finalité et toute autre information pertinente visée au paragraphe 2.

4. Les paragraphes 1, 2 et 3 ne s'appliquent pas lorsque, et dans la mesure où, la personne concernée dispose déjà de ces informations.

FORMULAIRE DE CONSENTEMENT -- « ESTIMABL3»
N°CSET 2017/2581 – ID-RCB 2017-A01779-44

Nom du patient : .... **Prénom : ....**

Il m'est proposé(e) de participer à une étude clinique organisée par Gustave Roussy, et intitulée ESTIMABL 3 "ESTIMAtion de l'impact de l'évidement ganglionnaire prophylactique du compartiment central du cou sur les résultats oncologiques des cancers différenciés de la thyroïde à Bas risque de récidive Loco-régionale".
En application des dispositions du Code de la santé publique, j'ai pris connaissance de la note d'information (***pages 1 à 8***). Il m'a été laissé le temps de poser toutes les questions que je souhaitais et toutes les questions posées ont reçu une réponse satisfaisante.
J'ai bien noté que je suis libre d'accepter ou de refuser de participer à l'étude et libre d'interrompre ma participation à tout moment, sans avoir à me justifier ni sans que cela n'ait de conséquence sur la suite et la qualité de mon suivi médical.
Je déclare être affilié(e) à un régime de sécurité sociale ou bénéficiaire d'un tel régime.
Les données médicales ainsi que le dossier médical me concernant resteront strictement confidentiels. Je n'autorise leur consultation que par Gustave Roussy, ses partenaires ou prestataires dûment habilités ainsi que par les autorités de santé. Je comprends que mon nom ne sera jamais mentionné dans les rapports consacrés à cette étude et que mon identité ne sera pas communiquée.
J'accepte le recueil de mes données personnelles ainsi que leur traitement informatique par le promoteur ou par des personnes ou sociétés agissant pour son compte. J'accepte que mes données rendues anonymes soient éventuellement transmises en dehors de France, voire dans un pays ne bénéficiant pas d'un niveau de protection des données équivalent à celui en vigueur au sein de l'Union Européenne (sous la responsabilité du Promoteur). Je suis informé(e) que je dispose d'un droit d'opposition à la transmission des données. Je peux exercer ce droit à tout moment et sous toutes formes, par l'intermédiaire du médecin investigateur qui contactera Gustave Roussy.
J'ai bien noté que si je retire mon consentement, les informations et les échantillons recueillis dans le cadre de cette étude seront conservés et analysés, sauf si j'exprime mon refus auprès du médecin investigateur.
J'ai été informé(e)

- Qu'une assurance a été souscrite par Gustave Roussy permettant de couvrir les dommages liés à l'étude conformément à l'art. L1121-10 du Code de la Santé Publique.
- Que cette étude sera conduite conformément à la législation française en vigueur et qu'elle a reçu l'autorisation de l'Agence Nationale de Sécurité du Médicament et des produits de santé et l'avis favorable du Comité de Protection des Personnes (CPP Ile de France 2).

J'accepte librement et volontairement de participer à l'étude ESTIMABL 3

**Cocher les cases appropriées en fonction de votre volonté (OUI/NON)**	**OUI**	**NON**
J'accepte librement et volontairement que les données collectées pourront être utilisées dans le cadre de futures recherches scientifiques en dehors de ce protocole de recherche clinique.	☐	☐
J'accepte librement et volontairement que les prélèvements biologiques qui m'ont été prélevés soient stockés pour être utilisés à des fins de recherche scientifique pour une durée indéterminée	☐	☐

**Partie à remplir par le patient**	**Partie à remplir par le médecin investigateur qui a recueilli le consentement**
Nom et prénom :	Nom et prénom :
Signature :	Signature :
Date :	Date :

(Original pour le médecin-investigateur, copie à remettre au patient)

GUSTAVE ROUSSY

CANCER CAMPUS
GRAND PARIS

/ DIRECTION DE LA RECHERCHE CLINIQUE

SERVICE PROMOTION DES ETUDES CLINIQUES

114, rue Edouard-Vaillant
94805 Villejuif Cedex - France

www.gustaveroussy.fr

Gustave Roussy promoteur

« la DRC est labellisée
Délégation à la Recherche
Clinique et à l'Innovation
par le Ministère de la Santé »
