# Supplementary material for: ESTIMation of the ABiLity of prophylactic central compartment neck dissection to modify outcomes in low-risk differentiated thyroid cancer: a prospective randomized trial
Source: Trials. 2023 Apr 28;24:298. doi: 10.1186/s13063-023-07294-0 (PMC10142499; doi:10.1186/s13063-023-07294-0)
Supplement: Supplementary file 2 — Additional file 2: Annex 2. [file 13063_2023_7294_MOESM2_ESM.docx]

# ANNEX 2: EQ-5D-3L

| Veuillez indiquer, pour chacune des rubriques suivantes, l’affirmation qui décrit le mieux votre état de santé aujourd’hui, en cochant la case appropriée. | |
| --- | --- |
|  |  |
| Mobilité |  |
| Je n’ai aucun problème pour me déplacer à pied | ❑ |
| J’ai des problèmes pour me déplacer à pied | ❑ |
| Je suis obligé(e) de rester alité(e) | ❑ |
|  |  |
| Autonomie de la personne |  |
| Je n’ai aucun problème pour prendre soin de moi | ❑ |
| J’ai des problèmes pour me laver ou m’habiller tout(e) seul(e) | ❑ |
| Je suis incapable de me laver ou de m’habiller tout(e) seul(e) | ❑ |
|  |  |
| Activités courantes *(exemples: travail, études, travaux domestiques, activités familiales ou loisirs)* |  |
| Je n’ai aucun problème pour accomplir mes activités courantes | ❑ |
| J’ai des problèmes pour accomplir mes activités courantes | ❑ |
| Je suis incapable d’accomplir mes activités courantes | ❑ |
|  |  |
| Douleurs / gêne |  |
| Je n’ai ni douleur ni gêne | ❑ |
| J’ai des douleurs ou une gêne modérée(s) | ❑ |
| J’ai des douleurs ou une gêne extrême(s) | ❑ |
|  |  |
| Anxiété / Dépression |  |
| Je ne suis ni anxieux(se) ni déprimé(e) | ❑ |
| Je suis modérément anxieux(se) ou déprimé(e) | ❑ |
| Je suis extrêmement anxieux(se) ou déprimé(e) | ❑ |
