# Supplementary material for: ESTIMation of the ABiLity of prophylactic central compartment neck dissection to modify outcomes in low-risk differentiated thyroid cancer: a prospective randomized trial
Source: Trials. 2023 Apr 28;24:298. doi: 10.1186/s13063-023-07294-0 (PMC10142499; doi:10.1186/s13063-023-07294-0)
Supplement: Supplementary file 3 — Additional file 3: Annex 3. [file 13063_2023_7294_MOESM3_ESM.docx]

# ANNEX 3: SF-36

**COMMENT REPONDRE :** Les questions qui suivent portent sur votre santé, telle que vous la ressentez. Ces informations nous permettront de mieux savoir comment vous vous sentez dans votre vie de tous les jours.

Veuillez répondre à **toutes les questions en** entourant le chiffre correspondant à la réponse choisie, comme il est indiqué. Si vous ne savez pas très bien comment répondre, choisissez la réponse la plus proche de votre situation.

**1. Dans l’ensemble, pensez-vous que votre santé est :**

*entourez la réponse de votre choix*

Excellente 1

Très bonne 2

Bonne 3

Médiocre 4

Mauvaise 5

**2. Par rapport au même jour de la semaine dernière, comment trouvez-vous votre état de santé en ce moment ?**

*entourez la réponse de votre choix*

Bien meilleur que la semaine dernière 1

Plutôt meilleur 2

À peu près pareil 3

Plutôt moins bon 4

Beaucoup moins bon 5

**3. Voici une liste d’activités que vous pouvez avoir à faire dans votre vie de tous les jours. Pour chacune d’entre elles indiquez si vous êtes limité(e) en raison de votre état de santé actuel.**

*entourez la réponse de votre choix, une par ligne*

| **Liste d’activités** | **Oui, beaucoup limité(e)** | **Oui, un peu limité(e)** | **Non, pas du tout limité(e)** |
| --- | --- | --- | --- |
| a. **Efforts physiques importants** tels que courir, soulever un objet lourd, faire du sport | 1 | 2 | 3 |
| b. **Efforts physiques modérés** tels que déplacer une table, passer l’aspirateur, jouer aux boules | 1 | 2 | 3 |
| c. Soulever et porter les courses | 1 | 2 | 3 |
| d. Monter **plusieurs étages** par l’escalier | 1 | 2 | 3 |
| e. Monter **un étage** par l’escalier | 1 | 2 | 3 |
| f. Se pencher en avant, se mettre à genoux, s’accroupir | 1 | 2 | 3 |
| g. Marcher **plus d’un km** à pied | 1 | 2 | 3 |
| h. Marcher **plusieurs centaines de mètres** | 1 | 2 | 3 |
| i. Marcher **une centaine de mètres** | 1 | 2 | 3 |
| j. Prendre un bain, une douche ou s’habiller | 1 | 2 | 3 |

**4. Au cours de cette dernière semaine, et en raison de votre état physique**

*entourez la réponse de votre choix, une par ligne*

|  | **En perma-nence** | **Très souvent** | **Quel-ques fois** | **Rare-ment** | **Jamais** |
| --- | --- | --- | --- | --- | --- |
| a. Avez-vous réduit le **temps passé** à votre travail  ou à vos activités habituelles ? | 1 | 2 | 3 | 4 | 5 |
| b. Avez-vous **accompli moins** de choses  que vous auriez souhaité ? | 1 | 2 | 3 | 4 | 5 |
| c. Avez-vous dû arrêter de faire **certaines** choses ? | 1 | 2 | 3 | 4 | 5 |
| d. Avez-vous eu des **difficultés** à faire votre travail ou toute autre activité ? (par exemple, cela vous a demandé un effort supplémentaire) | 1 | 2 | 3 | 4 | 5 |

**5. Au cours de cette dernière semaine, et en raison de votre état émotionnel (comme vous sentir triste, nerveux(se) ou déprimé(e))**
 *entourez la réponse de votre choix, une par ligne*

|  | **En perma-nence** | **Très souvent** | **Quel-ques fois** | **Rare-ment** | **Jamais** |
| --- | --- | --- | --- | --- | --- |
| a. Avez-vous réduit le **temps passé** à votre travail  ou à vos activités habituelles | 1 | 2 | 3 | 4 | 5 |
| b. avez-vous **accompli moins** de choses que  vous auriez souhaité | 1 | 2 | 3 | 4 | 5 |
| c. avez-vous eu des **difficultés** à faire ce que vous  aviez à faire avec autant de soin et d’attention  que d’habitude | 1 | 2 | 3 | 4 | 5 |

**6. Au cours de cette dernière semaine dans quelle mesure votre état de santé, physique ou émotionnel, vous a-t-il gêné(e) dans votre vie sociale et vos relations avec les autres, votre famille, vos amis, vos connaissances**

*entourez la réponse de votre choix*

Pas du tout 1

Un petit peu 2

Moyennement 3

Beaucoup 4

Enormément 5

**7. Au cours de cette dernière semaine, quelle a été l’intensité de vos douleurs physiques ?**

*entourez la réponse de votre choix*

Nulle 1

Très faible 2

Faible 3

Moyenne 4

Grande 5

Très grande 6

**8. Au cours de cette dernière semaine, dans quelle mesure vos douleurs physiques vous ont-elles limité(e) dans votre travail ou vos activités domestiques?**

*entourez la réponse de votre choix*

Pas du tout 1

Un petit peu 2

Moyennement 3

Beaucoup 4

Enormément 5

**9. Les questions qui suivent portent sur comment vous vous êtes senti(e) au cours de cette dernière semaine. Pour chaque question, veuillez indiquer la réponse qui vous semble la plus appropriée. Au cours de cette dernière semaine, y a-t-il eu des moments où :***entourez la réponse de votre choix, une par ligne*

|  | **En permanence** | **Très souvent** | **Souvent** | **Quelque fois** | **Rarement** | **Jamais** |
| --- | --- | --- | --- | --- | --- | --- |
| a. vous vous êtes senti(e) dynamique? | 1 | 2 | 3 | 4 | 5 | 6 |
| b. vous vous êtes senti(e) très nerveux(se)? | 1 | 2 | 3 | 4 | 5 | 6 |
| c. vous vous êtes senti(e) si découragé(e) que rien ne pouvait vous remonter le moral? | 1 | 2 | 3 | 4 | 5 | 6 |
| d. vous vous êtes senti(e) calme et détendu(e)? | 1 | 2 | 3 | 4 | 5 | 6 |
| e. vous vous êtes senti(e) débordant(e) d’énergie? | 1 | 2 | 3 | 4 | 5 | 6 |
| f. vous vous êtes senti(e) triste et abattu(e)? | 1 | 2 | 3 | 4 | 5 | 6 |
| 9. vous vous êtes senti(e) épuisé(e)? | 1 | 2 | 3 | 4 | 5 | 6 |
| h. vous vous êtes senti(e) heureux(se)? | 1 | 2 | 3 | 4 | 5 | 6 |
| i. vous vous êtes senti(e) fatigué(e)? | 1 | 2 | 3 | 4 | 5 | 6 |

**10. Au cours de cette dernière semaine, y a-t-il eu des moments où votre état de santé, physique ou émotionnel, vous a gêné(e) dans votre vie et vos relations avec les autres, votre famille, vos amis, vos connaissances ?**

*entourez la réponse de votre choix*

En permanence 1

Une bonne partie du temps 2

De temps en temps 3

Rarement 4

Jamais 5

**11. Indiquez pour chacune des phrases suivantes dans quelle mesure elles sont vraies ou fausses dans votre cas :**

*entourez la réponse de votre choix, une par ligne*

|  | **Totalement vrai** | **Plutôt vrai** | **Je ne sais pas** | **Plutôt fausse** | **Totalement fausse** |
| --- | --- | --- | --- | --- | --- |
| a. Je tombe malade plus facilement que les autres | 1 | 2 | 3 | 4 | 5 |
| b. Je me porte aussi bien que n’importe qui | 1 | 2 | 3 | 4 | 5 |
| c. Je m’attends à ce que ma santé se dégrade | 1 | 2 | 3 | 4 | 5 |
| d. Je suis en excellente santé | 1 | 2 | 3 | 4 | 5 |

**VEUILLEZ VERIFIER QUE VOUS AVEZ BIEN FOURNI UNE REPONSE POUR CHACUNE DES QUESTIONS. MERCI DE VOTRE COLLABORATION.**
