# Supplementary material for: ESTIMation of the ABiLity of prophylactic central compartment neck dissection to modify outcomes in low-risk differentiated thyroid cancer: a prospective randomized trial
Source: Trials. 2023 Apr 28;24:298. doi: 10.1186/s13063-023-07294-0 (PMC10142499; doi:10.1186/s13063-023-07294-0)
Supplement: Supplementary file 4 — Additional file 4: Annex 4. [file 13063_2023_7294_MOESM4_ESM.docx]

# ANNEX 4: FRENCH SPIELBERGER STAI QUESTIONNAIRE

| **Consigne :**  Un certain nombre de phrases que l'on utilise pour se décrire sont données ci-dessous.  Lisez chaque phrase, puis marquez d'une croix, parmi les quatre cases à droite, celle qui correspond le mieux à ce que vous ressentez **A L'INSTANT, JUSTE EN CE MOMENT**.  Il n'y a pas de bonnes ni de mauvaises réponses.  Ne passez pas trop de temps sur l'une ou l'autre de ces propositions, et indiquez la réponse qui décrit le mieux vos sentiments **actuels**. | | | | | | |
| --- | --- | --- | --- | --- | --- | --- |
|  |  | **(1)** | **(2)** | **(3)** | **(4)** |  |
|  |  | **Non** | **Plutôt non** | **Plutôt oui** | **Oui** |  |
| **1** | Je me sens calme………………………………………………….……………. | **□** | **□** | **□** | **□** |  |
| **2** | Je me sens en sécurité, sans inquiétude, en sûreté…………….….………. | **□** | **□** | **□** | **□** |  |
| **3** | Je suis tendue, crispée…………………………………………………………. | **□** | **□** | **□** | **□** |  |
| **4** | Je me sens surmenée……………………………………………………….….. | **□** | **□** | **□** | **□** |  |
| **5** | Je me sens tranquille, bien dans ma peau…………………………………… | **□** | **□** | **□** | **□** |  |
| **6** | Je me sens émue, bouleversée, contrariée………………………..………… | **□** | **□** | **□** | **□** |  |
| **7** | L'idée de malheurs éventuels me tracasse en ce moment…………………. | **□** | **□** | **□** | **□** |  |
| **8** | Je me sens contente…………….……………………………………………… | **□** | **□** | **□** | **□** |  |
| **9** | Je me sens effrayée……………………….…………………………………… | **□** | **□** | **□** | **□** |  |
| **10** | Je me sens à mon aise………………………………….………………….….. | **□** | **□** | **□** | **□** |  |
| **11** | Je sens que j'ai confiance en moi……………………….…………………….. | **□** | **□** | **□** | **□** |  |
| **12** | Je me sens nerveuse, irritable………………………………….……………... | **□** | **□** | **□** | **□** |  |
| **13** | J'ai la frousse, la trouille j'ai peur…………….………………………………... | **□** | **□** | **□** | **□** |  |
| **14** | Je me sens indécise……………………..……………………………………… | **□** | **□** | **□** | **□** |  |
| **15** | Je suis décontractée, détendue………………….………………….………… | **□** | **□** | **□** | **□** |  |
| **16** | Je suis satisfaite……………………..………………………………………….. | **□** | **□** | **□** | **□** |  |
| **17** | Je suis inquiète, soucieuse……………………………….……………………. | **□** | **□** | **□** | **□** |  |
| **18** | Je ne sais plus où j'en suis, je me sens déconcertée, déroutée………...…. | **□** | **□** | **□** | **□** |  |
| **19** | Je me sens solide, posée, pondérée, réfléchie…………….………………... | **□** | **□** | **□** | **□** |  |
| **20** | Je me sens de bonne humeur, aimable………………………………..…….. | **□** | **□** | **□** | **□** |  |
