# Supplementary material for: ESTIMation of the ABiLity of prophylactic central compartment neck dissection to modify outcomes in low-risk differentiated thyroid cancer: a prospective randomized trial
Source: Trials. 2023 Apr 28;24:298. doi: 10.1186/s13063-023-07294-0 (PMC10142499; doi:10.1186/s13063-023-07294-0)
Supplement: Supplementary file 5 — Additional file 5: Annex 5. [file 13063_2023_7294_MOESM5_ESM.docx]

|  |
| --- |
|  |
|  |
|  |
|  |
|  |

# ANNEX 5: Voice handicap index

Ce questionnaire a pour but d’évaluer les effets d’éventuelles modifications de votre voix sur votre vie quotidienne, au cours de la semaine passée. Entourer le chiffre qui correspond à votre expérience personnelle.

### Jamais Presque jamais Parfois Presque toujours Toujours

**0 1 2 3 4**

F1. On a du mal à m’entendre à cause de ma voix. 0 1 2 3 4

F2. On me comprend difficilement

dans un milieu bruyant. 0 1 2 3 4

F8. Mes problèmes de voix limitent ma vie

personnelle et sociale. 0 1 2 3 4

F9. Je me sens exclu(e) des conversations

à cause de ma voix. 0 1 2 3 4

F10. Mes problèmes de voix entraînent

une perte de revenu. 0 1 2 3 4

P3. Les gens me posent des questions sur ma voix. 0 1 2 3 4

P5. J’ai l’impression que je dois me forcer

physiquement pour parler. 0 1 2 3 4

P6. La clarté de ma voix est imprévisible. 0 1 2 3 4

E4. Mes problèmes de voix me contrarient. 0 1 2 3 4

E6. Je mes sens handicapé(e) à cause de ma voix. 0 1 2 3 4

Comment votre voix est-elle aujourd’hui ?

### Normale (bonne qualité) Un peu anormale Assez anormale Très anormale

Sur une échelle de 0 à 10, à quel degré votre problème de voix influence-t-il votre qualité de vie ?

#### Pas du tout Enormément

**0 1 2 3 4 5 6 7 8 9 10**
