# Supplementary material for: ESTIMation of the ABiLity of prophylactic central compartment neck dissection to modify outcomes in low-risk differentiated thyroid cancer: a prospective randomized trial
Source: Trials. 2023 Apr 28;24:298. doi: 10.1186/s13063-023-07294-0 (PMC10142499; doi:10.1186/s13063-023-07294-0)
Supplement: Supplementary file 6 — Additional file 6: Annex 6. [file 13063_2023_7294_MOESM6_ESM.docx]

# ANNEX 6: French SwalQoL

| **IMPORTANT**: Nous comprenons que vous avez plusieurs problèmes de santé. Il est parfois difficile de les séparer. Essayer de faire de votre mieux pour ne penser qu'à vos problèmes de déglutition. Merci pour vos efforts!  Les questions ci dessous concernent des troubles fréquement rencontrés dans les problèmes de déglutitions. Dans le dernier mois, avez vous rencontré les problèmes suivant?  *(n'entourer qu'un chiffre)* | | | | | | |
| --- | --- | --- | --- | --- | --- | --- |
|  | Toujours vrai | Souvent vrai | Parfois vrai | Rarement vrai | Pas vrai du tout |  |
| Vivre avec mes troubles de déglutition est difficile? | 1 | 2 | 3 | 4 | 5 |  |
| Mes problèmes de déglutition sont une gêne majeure dans ma vie? | 1 | 2 | 3 | 4 | 5 |  |
| Les questions ci dessous rapportent des plaintes que les patients avec des troubles de la déglutition décrivent. Dans le dernier mois, avez vous rencontré les problèmes suivants?  *(n'entourer qu'un chiffre)* | | | | | | |
|  | Toujours vrai | Souvent vrai | Parfois vrai | Rarement vrai | Pas vrai du tout |  |
| Presque tous les jours, je ne fais pas attention si je mange ou pas | 1 | 2 | 3 | 4 | 5 |  |
| Je prend plus de temps que les autres pour manger | 1 | 2 | 3 | 4 | 5 |  |
| J'ai rarement faim | 1 | 2 | 3 | 4 | 5 |  |
| Mon repas me prend beaucoup de temps | 1 | 2 | 3 | 4 | 5 |  |
| Je ne prends plus de plaisir à manger | 1 | 2 | 3 | 4 | 5 |  |

| Les questions ci dessous rapportent des signes que les patients avec des troubles de la déglutition décrivent. Dans le dernier mois, avez vous rencontré les problèmes suivant?  *(n'entourer qu'un chiffre)* | | | | | |
| --- | --- | --- | --- | --- | --- |
|  | Toujours | Souvent | Parfois | Rarement | Jamais |
| Je tousse | 1 | 2 | 3 | 4 | 5 |
| Je m'étouffe en mangeant des aliments | 1 | 2 | 3 | 4 | 5 |
| Je m'étouffe en buvant | 1 | 2 | 3 | 4 | 5 |
| J'ai une salive épaisse et / ou des glaires | 1 | 2 | 3 | 4 | 5 |
| J'ai envie de vomir | 1 | 2 | 3 | 4 | 5 |
| Je me racle la gorge | 1 | 2 | 3 | 4 | 5 |
| J'ai des problèmes pour macher | 1 | 2 | 3 | 4 | 5 |
| J'ai trop de salive ou de crachats | 1 | 2 | 3 | 4 | 5 |
| Je n'arrive pas à me dégager la gorge | 1 | 2 | 3 | 4 | 5 |
| Les aliments restent coincés dans ma gorge | 1 | 2 | 3 | 4 | 5 |
| Les aliments restent collés dans ma bouche | 1 | 2 | 3 | 4 | 5 |
| Les aliments ou les liquides ressortent par ma bouche | 1 | 2 | 3 | 4 | 5 |
| Les aliments ou les liquides ressortent par mon nez | 1 | 2 | 3 | 4 | 5 |
| Je n'arrive pas à tousser quand les aliments sont coincés | 1 | 2 | 3 | 4 | 5 |
|  | | | | | |

| Ensuite, répondre si'l vous plait sur des questions concernant votre alimentation et vos repas  *(n'entourer qu'un chiffre)* | | | | | |
| --- | --- | --- | --- | --- | --- |
|  | Tout à fait d'accord | D'accord | Incertain | Pas d'accord | Pas du tout d'accord |
| Le choix de mes aliments est difficile | 1 | 2 | 3 | 4 | 5 |
| Il est difficile de trouver une alimentation adaptée que j'aime | 1 | 2 | 3 | 4 | 5 |
| Dans le mois précédent, avez eu des problèmes de communication avec les autres à cause de vos problèmes de déglutition ?  *(n'entourer qu'un chiffre)* | | | | | |
|  | Tout le temps | La plupart du temps | Parfois | Quelque fois | Jamais |
| Les gens ont du mal à comprendre ce que je dis | 1 | 2 | 3 | 4 | 5 |
| C'est difficile pour moi de parler clairement | 1 | 2 | 3 | 4 | 5 |
| Les questions ci dessous rapportent des impressions que les patients avec des troubles de la déglutition décrivent. Dans le dernier mois, avez vous rencontré les problèmes suivant  *(n'entourer qu'un chiffre)* | | | | | |
|  | Toujours | Souvent | Parfois | Rarement | Jamais |
| J'ai peur d'étouffer en mangeant | 1 | 2 | 3 | 4 | 5 |
| J'ai peur d'avoir une pneumonie | 1 | 2 | 3 | 4 | 5 |
| J'ai peur de m'étouffer quand je bois | 1 | 2 | 3 | 4 | 5 |
| Je ne sais jamais si je vais étouffer | 1 | 2 | 3 | 4 | 5 |
|  | | | | | |

| Dans le mois dernier, avez vous ressenti les propositions suivantes à cause de vos problèmes de déglutition ?  *(n'entourer qu'un chiffre)* | | | | | |
| --- | --- | --- | --- | --- | --- |
|  | Toujours vrai | Souvent vrai | Parfois vrai | Rarement vrai | Pas vrai du tout |
| Ma déglutition me déprime | 1 | 2 | 3 | 4 | 5 |
| Je suis gêné(e) par ma déglutition | 1 | 2 | 3 | 4 | 5 |
| Je suis contrarié(e) par ma déglutition | 1 | 2 | 3 | 4 | 5 |
| Mes problèmes de déglutition sont frustrants | 1 | 2 | 3 | 4 | 5 |
| Je suis impatient de régler ce problème | 1 | 2 | 3 | 4 | 5 |
| Dans le mois dernier, avez vous eu des ennuis dans vos relations avec les autres à cause de vos problèmes de déglutition ?  *(n'entourer qu'un chiffre)* | | | | | |
|  | Tout à fait d'accord | D'accord | Incertain | Pas d'accord | Pas du tout d'accord |
| Je ne mange plus à l'extérieur | 1 | 2 | 3 | 4 | 5 |
| C'est difficile d'avoir une vie sociale | 1 | 2 | 3 | 4 | 5 |
| J'ai changé de travail et/ou de loisirs à cause de ces problèmes | 1 | 2 | 3 | 4 | 5 |
| Je ne profite plus des fêtes ou des vacances | 1 | 2 | 3 | 4 | 5 |
| Ma position vis à vis de ma famille ou des mes amis a changé | 1 | 2 | 3 | 4 | 5 |
|  | | | | | |

| Dans le mois dernier, avez vous eu les signes suivants?  *(n'entourer qu'un chiffre)* | | | | | | |
| --- | --- | --- | --- | --- | --- | --- |
|  | Tout le temps | La plupart du temps | Parfois | Quelque fois | Jamais |  |
| Je me sens faible | 1 | 2 | 3 | 4 | 5 |  |
| J'ai des problèmes pour dormir | 1 | 2 | 3 | 4 | 5 |  |
| Je suis fatigué(e) | 1 | 2 | 3 | 4 | 5 |  |
| Je me réveille la nuit | 1 | 2 | 3 | 4 | 5 |  |
| Je me sens épuisé(e) | 1 | 2 | 3 | 4 | 5 |  |
| **Quelqu'un vous a t'il aidé à remplir ce questionnaire?**  Non, je l'ai fait seul  Oui quelqu'un m'a aidé  **Si quelqu'un vous a aidé, comment l'a t'il fait?**  Il a lu les questions et écrit vos réponses A répondu aux questions pour vous?  A fait autre chose  Merci d'indiquer la date d'aujourd'hui  / /  Jour Mois Années | | | | | | |
