# Supplementary figures and images for: ESTIMation of the ABiLity of prophylactic central compartment neck dissection to modify outcomes in low-risk differentiated thyroid cancer: a prospective randomized trial
Source: Trials. 2023 Apr 28;24:298. doi: 10.1186/s13063-023-07294-0 (PMC10142499; doi:10.1186/s13063-023-07294-0)

# ANNEX 7 – Clavien-Dinco Classification of surgical complications


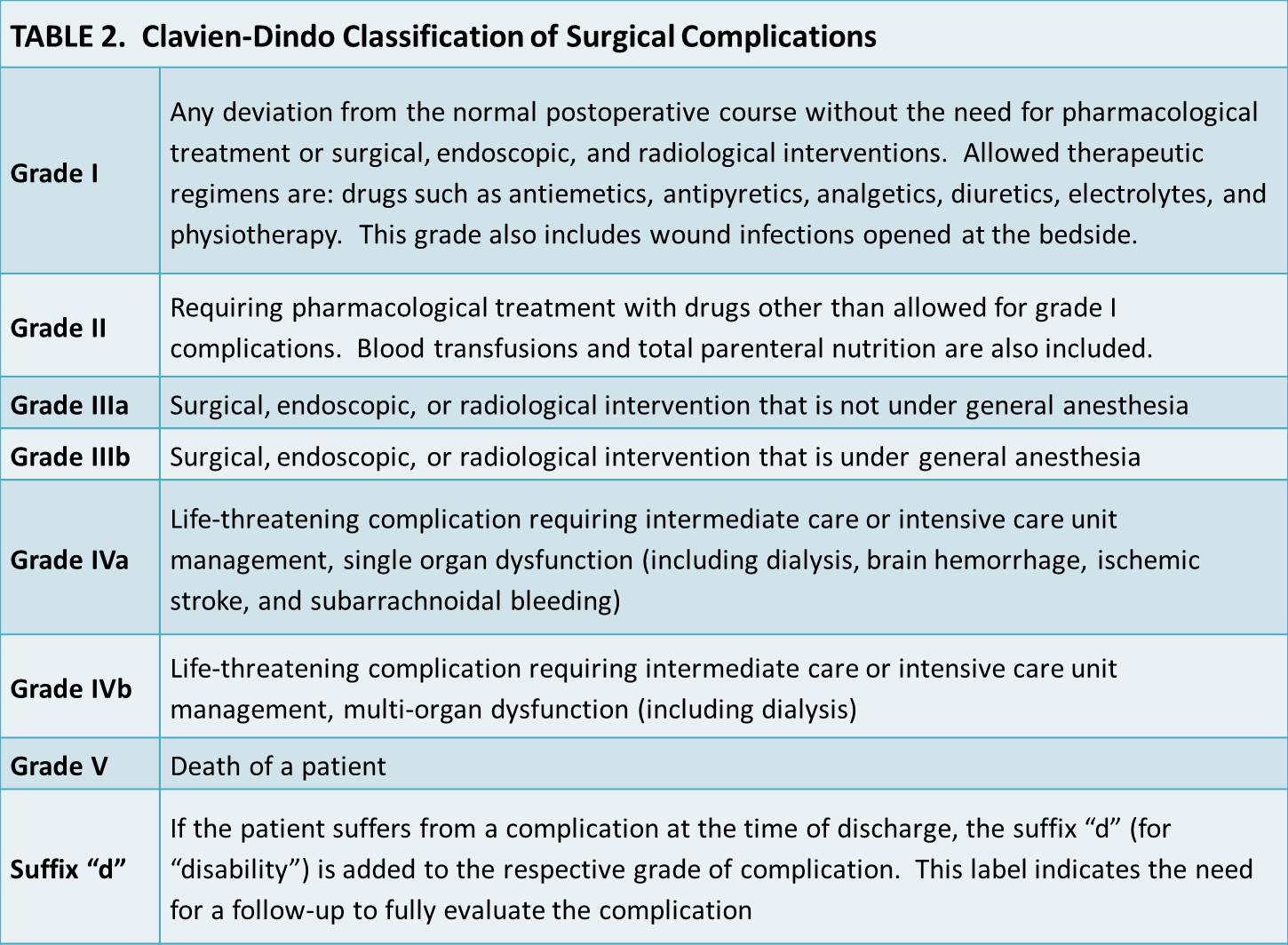

Supplement: Supplementary file 7 — Additional file 7: Annex 7. [file 13063_2023_7294_MOESM7_ESM.docx]
