# Supplementary material for: ESTIMation of the ABiLity of prophylactic central compartment neck dissection to modify outcomes in low-risk differentiated thyroid cancer: a prospective randomized trial
Source: Trials. 2023 Apr 28;24:298. doi: 10.1186/s13063-023-07294-0 (PMC10142499; doi:10.1186/s13063-023-07294-0)
Supplement: Supplementary file 8 — Additional file 8: Annex 8. [file 13063_2023_7294_MOESM8_ESM.zip › 13063_2023_7294_MOESM8_ESM.docx]

Translation of the Approval for Funding of the protocol ESTIMABL 03 by the French National Cancer Institute

National Cancer Institute (INCa)

Project Platform

Boulogne Billancourt, France, March 9, 2016

**References INCa**: 2016-janv001-BL/CFB

**Project Reference**: PHRC-K 15 – 182

**Theme**: Hospital Clincal Research Program, Oncology, « PHRC-K 15 »

**Reference**: Decision number DGOS/PF4/2014/349 December 19, 2014 relating to the programs on health and healthcare research for 2015

Dear Colleague,

Regarding the above cited project submitted for evaluation, I have the pleasure to inform you that the project « ESTIMABL 03 : **ESTIM**ation of the **AB**i**L**ity of prophylactic central compartment neck dissection to modify outcomes in low-risk differentiated thyroid cancer: A Prospective Randomized Trial” has been accepted for funding after going through the selection process delegated to the National Cancer Institute (INCa) by the General Direction of Health Care (DGOS). Financing will be distributed in the form of a grant under the auspices of the MERRI funds (teaching, research and innovation missions).

**I confirm that the sum granted to your project is 629 434 euros.**

The first part of the grant will be credited to you and announced through the first publication of the DGOS as part of the hospital research funding campaign for 2016.

The later allotments will be credited to you as the project is implemented. There are 5 phases of implementation, each associated with 5 parts of the grant. Each subsequent phase N+1 can only be financed after the previous phase N has been completed.

The necessary documents for each phase are detailed in an attached document, as are the formats for each document.

The INCa does not impose a timeline or deadlines for the phases of implementation of the study. After the necessary documents are received by the INCa and validated, the corresponding part of the grant will be allotted.

The necessary documents may only be transmitted electronically at : [suiviPHRC-K@institutcancer.fr](mailto:suiviPHRC-K@institutcancer.fr).

The transmission of the documents may only be done by the legal administrator of the hospital, healthcare group, healthcare center or healthcare coordinator and funds manager or by an authorized representative, with a copy sent to the principal investigator. Each document also requires the name and contact information of the legal administator (or authorized representative) and the principal investigator. Documents that do not include this necessary information will be considered void.

You are required to follow the requirements of the INCa :

- In the instance where the opinion of a Patient Protection Comite is required, it must be obtained
- Any modification of the protocol must be submitted for authorization to the INCa
- L’INCa organizes a scientific follow-up of all of the Cancer projects via an auto-questionnaire and via annual reporting meetings
- Each investigator-coordinator agrees to attend the meetings arranged by the INCa and to present the advancement of their projects
- Scientific publications resulting from research funded must mention that the grant was allotted under the following number « INCa-DGOS-9823. »
- The INCa retains the right to make the results of the study public by publishing the results on the website.

I would be grateful if you would be particularly attentive in following the procedures that will aid in implementing your project and to keep us informed as to any difficulties you may encounter when following these procedures.

I thank you for your trust in us, and please accept, Dear Colleague, the expression of my distinguished consideration.

Professor Agnès Buzin, President (signed by Thierry Breton, Interim President of the INCa)

Attached documents :

- Synthesis from the International Comite for Clinical Research in Oncology
- File for follow-up of the funded projects
- Schematic - grant allottment in 2015

Copy sent to :

Professor Gilles Vassal, Director of Clincial Research and Innovation
