# Supplementary material for: ESTIMation of the ABiLity of prophylactic central compartment neck dissection to modify outcomes in low-risk differentiated thyroid cancer: a prospective randomized trial
Source: Trials. 2023 Apr 28;24:298. doi: 10.1186/s13063-023-07294-0 (PMC10142499; doi:10.1186/s13063-023-07294-0)
Supplement: Supplementary file 8 — Additional file 8: Annex 8. [file 13063_2023_7294_MOESM8_ESM.zip › 13063_2023_7294_MOESM8_ESM.pdf]

INSTITUT NATIONAL DU CANCER

Plateforme Appel à projets

COPIE

Institut Gustave Roussy
Docteur Dana HARTL
114 Rue Edouard Vaillant
94805 – VILLEJUIF Cedex

Boulogne Billancourt, le 09 MARS 2016

Nos références : 2016-janv001-BL/CFB

Référence du Projet : PHRC-K 15 -182

Objet : Programme Hospitalier de Recherche Clinique, Volet Cancérologique « PHRC-K 15 »

Référence : INSTRUCTION N° DGOS/PF4/2014/349 du 19 décembre 2014 relative aux programmes de recherche sur les soins et l'offre de soins pour l'année 2015.

Chère Consoeur,

Dans le cadre de l'appel à projets visé en objet, j'ai le plaisir de vous annoncer que le projet : **«ESTIMABL 03 : ESTIMAtion de l'impact de l'évidement ganglionnaire prophylactique du compartiment central du cou sur la survie sans récidive des cancers différenciés de la thyroïde à Bas risque de récidive Loco-régionale »**, a été retenu au terme du processus de sélection confié à l'INCa par la DGOS. Le financement est alloué par la DGOS sous forme de dotation au titre des missions d'enseignement, de recherche, de référence et d'innovation (MERRI).

Je vous confirme que le financement de votre projet est de 629 434 €uros.

La première tranche des crédits sera notifiée dans la première CIRCULAIRE de la DGOS relative à la campagne tarifaire 2016 des établissements de santé.

Les tranches suivantes seront déléguées en fonction de la mise en œuvre effective de votre projet. Le déroulement d'un projet est identifié en 5 phases, associées à 5 tranches de financement. Le versement de la tranche de financement pour la phase N+1 est conditionné à la production des éléments qui finalisent la phase N.

Les éléments à produire à la finalisation de chaque phase en vue de l'obtention d'une tranche de financement sont précisés dans la fiche de suivi ci-jointe. Le format des documents à transmettre y est également précisé.

INSTITUT NATIONAL DU CANCER

Il n'y a pas de calendrier imposé pour la communication des éléments de suivi par les porteurs de projets. Dès lors que les éléments transmis à l'INCa sont validés, le versement de la tranche de financement correspondante sera effectué dans le cadre de la circulaire budgétaire subséquente.
La transmission des éléments de suivi doit se faire uniquement par voie électronique, selon les indications portées sur la fiche de suivi : suiviPHRC-K@institutcancer.fr
La transmission doit être faite par le responsable légal de l'établissement de santé, du GCS, de la maison de santé ou du centre de santé coordonnateur du projet (et gestionnaire des fonds) ou par son représentant dûment habilité, en mettant en copie le porteur du projet. Les coordonnées du responsable légal (ou de son représentant) et du porteur de projet sont portées sur chaque fiche de suivi. Le respect de ces dispositions conditionne la recevabilité des éléments transmis.

De plus, vous devrez tenir compte des spécificités de suivi exigées par l'INCa :

- **lorsque l'avis d'un Comité de Protection des Personnes (CPP) est requis, l'avis des comités de patients doit être obtenu.**
- **Toute modification d'un protocole initial est soumise à l'INCa.**
- **Pour remplir les objectifs des Plans Cancer, l'INCa conduit également un suivi scientifique des projets Cancer à travers une enquête par auto-questionnaire et des séminaires de restitution annuels.**
- **chaque investigateur coordonnateur s'engage à participer aux réunions organisées par l'INCa pour présenter l'état d'avancement des projets.**
- **les publications scientifiques résultant des travaux bénéficiant de ce financement devront mentionner ce soutien accompagné du numéro suivant : "INCa-DGOS_9823".**
- **L'INCa se réserve le droit de rendre public le rapport final des projets sur son site internet.**

Je vous serai reconnaissante d'accorder la plus grande attention à ces procédures qui doivent permettre d'aider à la conduite de votre projet et, de nous tenir informés des difficultés éventuelles que vous pourriez rencontrer dans la mise en œuvre de ces dispositions.

Je vous remercie de votre confiance et, vous prie d'agréer, Chère Consoeur, l'expression de ma considération distinguée.

Thierry BRETON
Président par intérim
INSTITUT NATIONAL DU CANCER

Pr. Agnès BUZYN
Présidente

Pièces jointes :

- **Arguments de synthèse du Comité International de Recherche Clinique en cancérologie**
- **Fiche de suivi des projets retenus**
- **Schéma-versement tranche 2015**

Copie :

- **Professeur Gilles VASSAL - Directeur de la recherche clinique et de l'innovation.**
