# Supplementary material for: ESTIMation of the ABiLity of prophylactic central compartment neck dissection to modify outcomes in low-risk differentiated thyroid cancer: a prospective randomized trial
Source: Trials. 2023 Apr 28;24:298. doi: 10.1186/s13063-023-07294-0 (PMC10142499; doi:10.1186/s13063-023-07294-0)
Supplement: Supplementary file 9 — Additional file 9: Annex 9. [file 13063_2023_7294_MOESM9_ESM.pdf]

Ethics Committee

Paris Region (Ile de France)

President: Marie-France MAMZER-BRUNEEL

Vice-president: Jean-Bernard CHARPENTIER

Secretaires: Pierre COLONNA, Jean-Louis BRESSON, Stéphane DONNADIEU

Members: C. ARDIOT, C. BADOUAL, C. BALLOUARD, M.-C. BARET, J.-L. BRESSON, C. BROISSAND, J.-B. CHARPENTIER, B. DEBAECKER, M. DE FALLOIS, N. LALLMAHAMOOD, M.-F. MAMZER-BRUNEL, P. VAN ES

Secretary: Nora VESTRIS

Dr Camille JANIN
DRC
114, rue Edouard Vaillant
94805 Villejuif

Paris, February 13, 2018

Research project number 2017-11-03 **ID-RCB/EUDRACT : 2017-A01779-44**

The research project entitled « Estimation of the AbiLity of prophylactic central compartment neck dissection to modify outcomes in low-risk differentiated thyroid cancer, » presented by Camille JANIN, was evaluated by the ethics committee on September 14th, 2017.

Insurance: SHAM, Contract number 124895

Investigator in France: Dr Dana Hartl, Gustave Roussy Hospital

Promotor: Institut Gustave Roussy

The information relative to this trial was examined during the meeting held on **February 5th, 2018**, based on the following elements:

- The administrative file containing:
  - The request letter, dated and signed
  - The request form, dated and signed
  - The additional document requested by the ethics committee, dated and signed
- The file concerning the research containing:
  - The research protocol dated and signed, version 1.0 dated September 13th, 2017
  - The summary of the French version of the protocol version 1.0 dated September 13th, 2017
  - The patient information document and the patient consent form version 1.0 dated September 13th, 2017
  - A copy of the certificate of insurance

The justification of the adequateness of the human, material and technical means, version 1.0 dated September 13th, 2017
The list of principal investigators version 1.0 dated September 13th, 2017 and January 24th, 2018
The curriculum vitae of the principal investigators in each center

The response to our remarks made on November 14th, 2017
The list of the principal investigators version 1.5 dated november 28th 2017 and their corresponding curriculum vitae
The patient information document and the patient consent form version 1.1 dated November 14th, 2017
The response to our remarks made on December 7th, 2017
The patient information document and the patient consent form version 1.2 dated January 5th, 2018

Those who participated in the deliberation (identity and quality of members, category): list on the last page.

After having verified that the research was conform with the article of the Public Health Code L-1121-1 and L-1123-7 defining the conditions for validity of research implicating the human person category 1°, the Comity adopts the following deliberation :

FAVORABLE OPINION

Pr Marie-France MAMZER-BRUNEEL
President of the Ethics Committee
Paris Region (Ile de France 2)

(STAMP)
Ethics Committee
Paris Region (Ile de France 2)
149, rue de Sèvres
75743 Paris cedex 15
Phone : 01 42 19 26 88
Fax : 01 44 49 45 06

PARIS REGION (ILE DE FRANCE 2)

President: M.-F. MAMZER-BRUNEEL
Vice-president: J-B CHARPENTIER
Secretaires: P. COLONNA
J-L BRESSON
S. DONNADIEU

MEETING ON FEBRUARY 5TH 2018

PERSONS QUALIFIED CONCERNING BIOMEDICAL RESEARCH:

-Dr MAMZER-BRUNEEL Marie-France	Present	Full member
-Dr DONNADIEU Stéphane	Present	Full member
-Pr COLONNA Pierre	Present	Full member
-Pr BRESSON Jean-Louis*	Present	Full member (pediatrics)
-Pr BADOUAL Cécile	Excused	Alternate member
-Dr JANNOT Anne-Sophie	Present	Alternate member
-vacant position		
-vacant position		

GENERAL PRACTITIONERS:

-Dr VAN ES Philippe	Present	Full member
-vacant position		

PHARMACISTS:

-Mrs BEAUSSIER Hélène	Excused	Full member
-Mrs BROISSAND Christine	Present	Alternate member

NURSES:

-Mrs BARET Marie-Christine	Excused	Full member
-vacant position		

PERSONS QUALIFIED IN ETHICS:

-vacant position
-vacant position

PSYCHOLOGISTS:

-Mr BALLOUARD Christian	Present	Full member
-Mr LALLMAHAMOOD Nizaar	Excused	Alternate member

PERSONS QUALIFIED IN THE SOCIAL DOMAIN:

-vacant position
-vacant position

PERSONS QUALIFIED IN THE JUDICIAL DOMAIN:

-Mrs de FALLOIS Manon	Present	Full member
-Mr MARTINET Eric	Excused	Alternate member
-2 vacant positions		

Research project number 2017-11-03 ID-RCB/EUDRACT : 2017-A01779-44

ACCREDITED ASSOCIATIONS OF PATIENTS OR CLIENTS OF THE HEALTH SYSTEM:

-Mrs ARDIOT Chantal	Present	Full member
-Mr CHARPENTIER Jean-Bernard	Present	Full member
-Mrs DEBAECKER Blanche	Excused	Alternate member
-Mrs DELSARTE Nicole	Present	Alternate member

*(competents in the domain of biostatistics and epidemiology)
