# Supplementary material for: ESTIMation of the ABiLity of prophylactic central compartment neck dissection to modify outcomes in low-risk differentiated thyroid cancer: a prospective randomized trial
Source: Trials. 2023 Apr 28;24:298. doi: 10.1186/s13063-023-07294-0 (PMC10142499; doi:10.1186/s13063-023-07294-0)
Supplement: Supplementary file 10 — Additional file 10: Annex 10. [file 13063_2023_7294_MOESM10_ESM.pdf]

sham
PARTAGEONS PLUS QUE L'ASSURANCE

# ATTESTATION D'ASSURANCE

=-=-=-=-=-=-=-=-=-=-=-=-=-=-=-=-=

**RESPONSABILITÉ CIVILE**
**PROMOTEUR DE RECHERCHES INTERVENTIONNELLES relevant de l'article L 1121-1, 1° du Code de la santé publique**

-------------------------------------------------------------------

*(Loi n°2012-300 du 5 mars 2012 et textes d'application subséquents)*

SOCIÉTÉ HOSPITALIÈRE D'ASSURANCES MUTUELLES
18, rue Édouard Rochet - 69372 LYON CEDEX 08

-----------------------------------------------------------------------------

atteste que **INSTITUT DE CANCEROLOGIE**
**GUSTAVE ROUSSY**
**114 RUE EDOUARD VAILLANT**
**94805 VILLEJUIF CEDEX**

A souscrit sous le n° **124895** un contrat d'assurance de la Responsabilité Civile Promoteur d'une Recherche interventionnelle relevant de l'article L 1121-1, 1° du Code de la santé publique conforme aux dispositions de l'article R 1121-4 du même code, afin de couvrir les obligations mises à leur charge en application de l'article L.1121-10 du même Code.

**« CSET 2017/2581 – ESTIMABL3 – Estimation de l'impact de l'évidement ganglionnaire prophylactique du compartiment central du cou sur les résultats oncologiques des cancers différenciés de la thyroïde à Bas risque de récidive Loco-régionale » (Dr Dana HARTL) – enregistrée sous le numéro : 2017-A01779-44**

Dates prévisionnelles de début et de fin de la recherche : **01/09/2017 – 31/08/2025** (soit 96 mois)

Nombre prévisionnel de personnes qu'il est prévu d'inclure : **1000 patients**

**La garantie s'exerce pour les recherches réalisées exclusivement en France métropolitaine et dans les départements et territoires d'Outre-mer**.

La présente attestation ne constitue toutefois qu'une présomption d'assurance à la charge de la Société avant validation par les autorités compétentes.

Fait et Certifié, à LYON, 06/07/2017

Philippe BIDARD
Souscription et vie des contrats
Direction Etablissements Publics de santé

SOCIÉTÉ HOSPITALIÈRE D'ASSURANCES MUTUELLES
18 rue Édouard Rochet
69372 LYON
Cedex 08
